# Preparation for a first-in-man lentivirus trial in patients with cystic fibrosis

**DOI:** 10.1136/thoraxjnl-2016-208406

**Published:** 2016-11-16

**Authors:** Eric W F W Alton, Jeffery M Beekman, A Christopher Boyd, June Brand, Marianne S Carlon, Mary M Connolly, Mario Chan, Sinead Conlon, Heather E Davidson, Jane C Davies, Lee A Davies, Johanna F Dekkers, Ann Doherty, Sabrina Gea-Sorli, Deborah R Gill, Uta Griesenbach, Mamoru Hasegawa, Tracy E Higgins, Takashi Hironaka, Laura Hyndman, Gerry McLachlan, Makoto Inoue, Stephen C Hyde, J Alastair Innes, Toby M Maher, Caroline Moran, Cuixiang Meng, Michael C Paul-Smith, Ian A Pringle, Kamila M Pytel, Andrea Rodriguez-Martinez, Alexander C Schmidt, Barbara J Stevenson, Stephanie G Sumner-Jones, Richard Toshner, Shu Tsugumine, Marguerite W Wasowicz, Jie Zhu

**Affiliations:** 1 Department of Gene Therapy, National Heart and Lung Institute, Imperial College London, London, UK; 2 UK Cystic Fibrosis Gene Therapy Consortium, Oxford, UK; 3 Department of Pediatric Pulmonology, Laboratory of Translational Immunology, Wilhelmina Children's Hospital, University Medical Centre, Utrecht, The Netherlands; 4 Centre for Genomic and Experimental Medicine, IGMM, University of Edinburgh, Edinburgh, UK; 5 Lung Pathology Unit, Department of Airway Disease Infection, NHLI, Imperial College London, London, UK; 6 Laboratory for Molecular Virology and Gene Therapy, Department of Pharmaceutical and Pharmacological Sciences, KU Leuven, Brussels, Belgium; 7 Gene Medicine Research Group, NDCLS, John Radcliffe Hospital, Oxford, UK; 8 ID Pharme Co. Ltd. (DNAVEC Center), Tsukuba, Japan; 9 Roslin Institute & R(D)SVS, University of Edinburgh, Midlothian, UK; 10 Fibrosis Research Group, Inflammation, Repair & Development Section, National Heart and Lung Institute, Sir Alexander Fleming Building, Imperial College, London, UK; 11 Ave Leopold Wiener, Brussels, Belgium

**Keywords:** Cystic Fibrosis

## Abstract

We have recently shown that non-viral gene therapy can stabilise the decline of lung function in patients with cystic fibrosis (CF). However, the effect was modest, and more potent gene transfer agents are still required. Fuson protein (F)/Hemagglutinin/Neuraminidase protein (HN)-pseudotyped lentiviral vectors are more efficient for lung gene transfer than non-viral vectors in preclinical models. In preparation for a first-in-man CF trial using the lentiviral vector, we have undertaken key translational preclinical studies. Regulatory-compliant vectors carrying a range of promoter/enhancer elements were assessed in mice and human air–liquid interface (ALI) cultures to select the lead candidate; cystic fibrosis transmembrane conductance receptor (CFTR) expression and function were assessed in CF models using this lead candidate vector. Toxicity was assessed and ‘benchmarked’ against the leading non-viral formulation recently used in a Phase IIb clinical trial. Integration site profiles were mapped and transduction efficiency determined to inform clinical trial dose-ranging. The impact of pre-existing and acquired immunity against the vector and vector stability in several clinically relevant delivery devices was assessed. A hybrid promoter hybrid cytosine guanine dinucleotide (CpG)- free CMV enhancer/elongation factor 1 alpha promoter (hCEF) consisting of the elongation factor 1α promoter and the cytomegalovirus enhancer was most efficacious in both murine lungs and human ALI cultures (both at least 2-log orders above background). The efficacy (at least 14% of airway cells transduced), toxicity and integration site profile supports further progression towards clinical trial and pre-existing and acquired immune responses do not interfere with vector efficacy. The lead rSIV.F/HN candidate expresses functional CFTR and the vector retains 90–100% transduction efficiency in clinically relevant delivery devices. The data support the progression of the F/HN-pseudotyped lentiviral vector into a first-in-man CF trial in 2017.

**Key messages**

**What is the key question?**

- Is a lentiviral vector, which was pseudotyped to achieve efficient gene transfer into airway epithelial cells, suitable for progression into a first-in-man gene therapy trial in patients with cystic fibrosis (CF)?

**What is the bottom line?**

- The data support the progression of the F/HN-pseudotyped lentiviral vector into a first-in-man CF trial in 2017 for which funding has been obtained.

**Why read on?**

- In contrast to other viral vectors, lentiviral vectors hold substantial promise for the development of gene therapy for a range of diseases, including chronic conditions due to their high efficacy, duration of expression and the fact that pre-existing and acquired immune responses do not interfere with vector efficacy on repeated administration.

## Introduction

Our ongoing efforts to improve pulmonary gene transfer for the treatment of lung diseases such as cystic fibrosis (CF) have led to the assessment of a lentiviral vector (simian immunodeficiency virus (SIV)) pseudotyped with the Sendai virus (SeV) envelope proteins F and HN (rSIV.F/HN).^1^ The latter contribute significantly to the high transduction efficiency of SeV-based vectors in the airway epithelium.^2^

We have previously shown that F/HN-pseudotyped SIV vector produced gene expression in the lungs and nose of mice for the duration of their lifetime (∼2 years). Further, this expression was at least 2-log orders higher than our lead non-viral formulation recently shown to produce significant effects in the lungs of patients with CF. Repeated daily administration led to a cumulative dose-related increase in gene expression, while repeated monthly administration to murine lower airways was feasible without loss of gene expression. There was no evidence of chronic toxicity during a 2-year study period and F/HN-pseudotyped SIV led to persistent gene expression in human differentiated airway cultures and human lung slices and transduced freshly obtained primary human airway epithelial cells.^3^ ^4^ In contrast to other pseudotypes, such as vesicular stomatitis virus glycoprotein (VSV-G)^5^ or GP64,^6^ the F/HN pseudotype does not require coadministration of compounds that open tight junctions or inhibit ciliary beating,^3^ ^4^ likely making the vector more acceptable for clinical translation in the context of CF chronic pulmonary bacterial infection.

It has been suggested that self-inactivating (SIN) lentiviral vectors may carry less risk of insertional mutagenesis due to inactivation of the promoter/enhancer properties in the long-terminal repeat (LTR) which were responsible for proto-oncogene transactivation in some γ-oncoretroviral vector trials.^7^ In the most studied context of haematopoetic stem cells (HSCs), differences in insertion site (IS) profiles between γ-oncoretroviral and lentiviral vectors have favoured the safety profile of the latter.^7^ ^8^ Further, recent studies with γ-oncoretroviral^9^ and lentiviral^10^ vectors in patients with Wiskott–Aldrich syndrome have allowed, for the first time, direct comparison of safety and efficacy of these vectors in man. Until now, the data support an improved safety profile of lentiviral vectors in the context of HSC transduction. Further, clinical studies in patients with metachromatic leukodystrophy^11^ and Parkinson's disease^12^ have not raised any safety concerns for lentiviral vectors, although longer follow-up is required.

To catalyse translation of the lentiviral vector platform into clinic, we have now selected the clinical lead candidate by generating pharmacopoeia-compliant producer plasmids and cGMP-compliant vector production methods (Virus Production paper; in preparation) and comparing several promoter/enhancer elements in both integrase-competent (IC) and integrase-defective (ID)^13^ vectors in mouse lung and ex vivo human models. Further, we have (a) mapped integration sites, (b) characterised transduced cell types, (c) assessed acute toxicity, (d) determined the effects of pre-existing immunity on transduction efficiency and toxicity, (e) assessed CFTR function of our lead vector and (f) quantified vector stability in delivery devices suitable for a first-in-man trial. We propose that this combined body of data supports the progression of the rSIV.F/HN vector into a first-in-man CF clinical trial.

## Materials and methods

See online supplementary material.

### Statistical analysis

**Figure 1:**
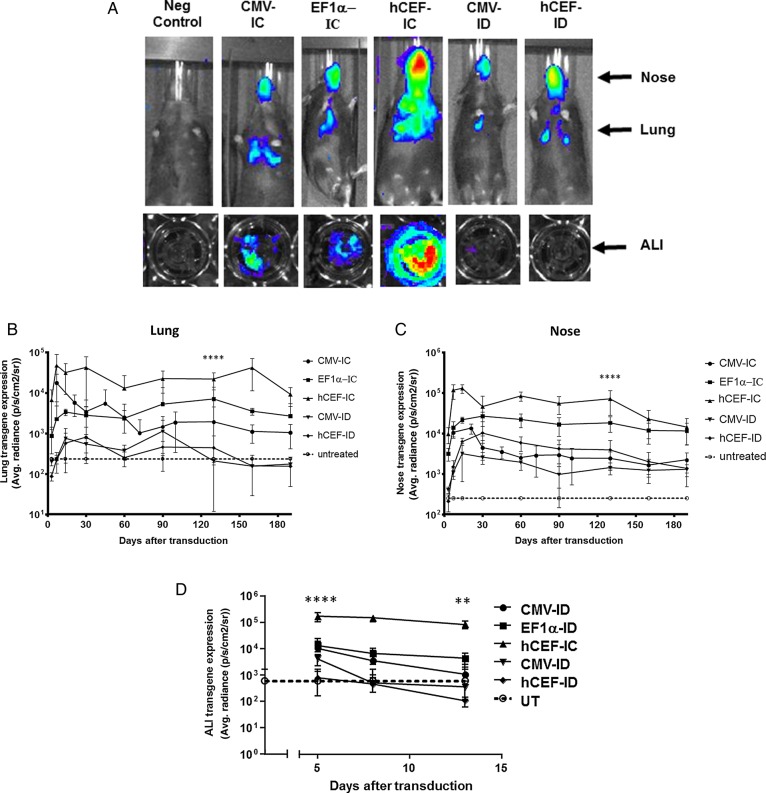
Selection of lead candidate vector. Mice and human air–liquid interface (ALI) cultures were transduced with five different lentiviral vector configurations by nasal instillation: integrase-competent (IC) vectors carrying the human elongation factor 1α (short) promoter (EF1α),^23^ a ubiquitous regulatory element which has previously been used in the context of lentivirus-mediated gene transfer,^48^ an in-house, synthetic chimeric promoter/enhancer consisting of CpG-depleted versions of the human EF1α (short) promoter and the human cytomegalovirus (CMV) enhancer (hCEF)^24^ and the original CMV-based construct, as well as integrase-defective (ID) vectors carrying the CMV promoter and hCEF promoter/enhancers (6–30E7 TU/mouse or ALI, n=6–10 mice/group, n=4 ALI/group). All vectors carried a luciferase reporter gene for quantification of gene expression by bioluminescence imaging. Negative control mice and ALIs remained untreated (UT). Gene expression was quantified in the lungs and nose of mice and in ALIs. Photon emission adjusted for differences in vector titre. (A) Representative images of transduced and UT mice and ALIs, (B) quantification of photon emission in murine lungs, (C) quantification of photoemission in murine nose and (D) in human ALIs. (B–D) Reference UT control values are shown as a dotted line (lung control: 182±6 p/s/cm^2^/sr, nose control: 200±10 p/s/cm^2^/sr, ALI control 598±1080 p/s/cm^2^/sr). For each group, the mean±SEM are shown. ****p<0.001 in lung and nose comparing hCEF-ID with all other vectors in mice (ANOVA followed by Tukey post hoc test), ***p<0.005 comparing hCEF-IC with UT ALI controls (Mann–Whitney).

All analyses were performed using GraphPad Prism6. Parametric and non-parametric data distributions were assessed with the Kolmogorov-Smirnov normality test. Multiple group and two-group comparisons were performed using appropriate statistical tests for specific data sets (see details in individual figure legends). In figure 1B, C, cross-sectional statistical analysis was performed on a selected time point and, therefore, no adjustments for longitudinal correlations were made. The null hypothesis was rejected at p<0.05.

## Results

### Selection of lead candidate vector from murine and human tissue studies

Our previously published studies were carried out with IC vectors using the cytomegalovirus (CMV) immediate early promoter/enhancer to regulate gene expression.^3^ ^4^ Here, we compared the CMV promoter/enhancer with the eukaryotic elongation factor 1α (EF1α) promoter and a chimeric regulatory element consisting of the human CMV enhancer coupled to the EF1α promoter (hCEF), with the aim to select the most efficient construct for progression into clinical trials (see online supplementary material for details on vector production). All experiments were performed with the maximum feasible volume/dose and vector titres for each configuration (see online supplementary table S1 for details). The hCEF-IC vector configuration achieved the highest and most persistent gene expression in the murine lung and nose (p<0.001) when compared with all other constructs 130 days after transduction (figure 1A–C). This ∼3 month time point was predefined for cross-sectional analysis, based on previous data. The ID vector configurations did not differ from untreated (UT) controls.

10.1136/thoraxjnl-2016-208406.supp1 Supplementary data

Air–liquid interface (ALI) cultures were also transduced with the five vector configurations ranging from 6 to 30E7 TU/ALI (n=4/group). The ID vector configuration did not differ from UT controls, but all three IC configurations lead to significant (p<0.005 for CMV-IC and EF1α-IC, and p<0.0001 for hCEF-IC at day 5 when compared with controls) levels of gene expression on day 5, which persisted for the hCEF-IC vector to day 14 (p<0.01). Consistent with data from mice, the hCEF-IC configuration achieved the highest and most persistent gene expression in ALIs (figure 1A, D) and was consequently selected as the lead candidate for progression into clinical trials with the designation rSIV.F/HN-hCEF from here onwards.

### Gene expression occurs in relevant airway epithelial cells

**Figure 2:**
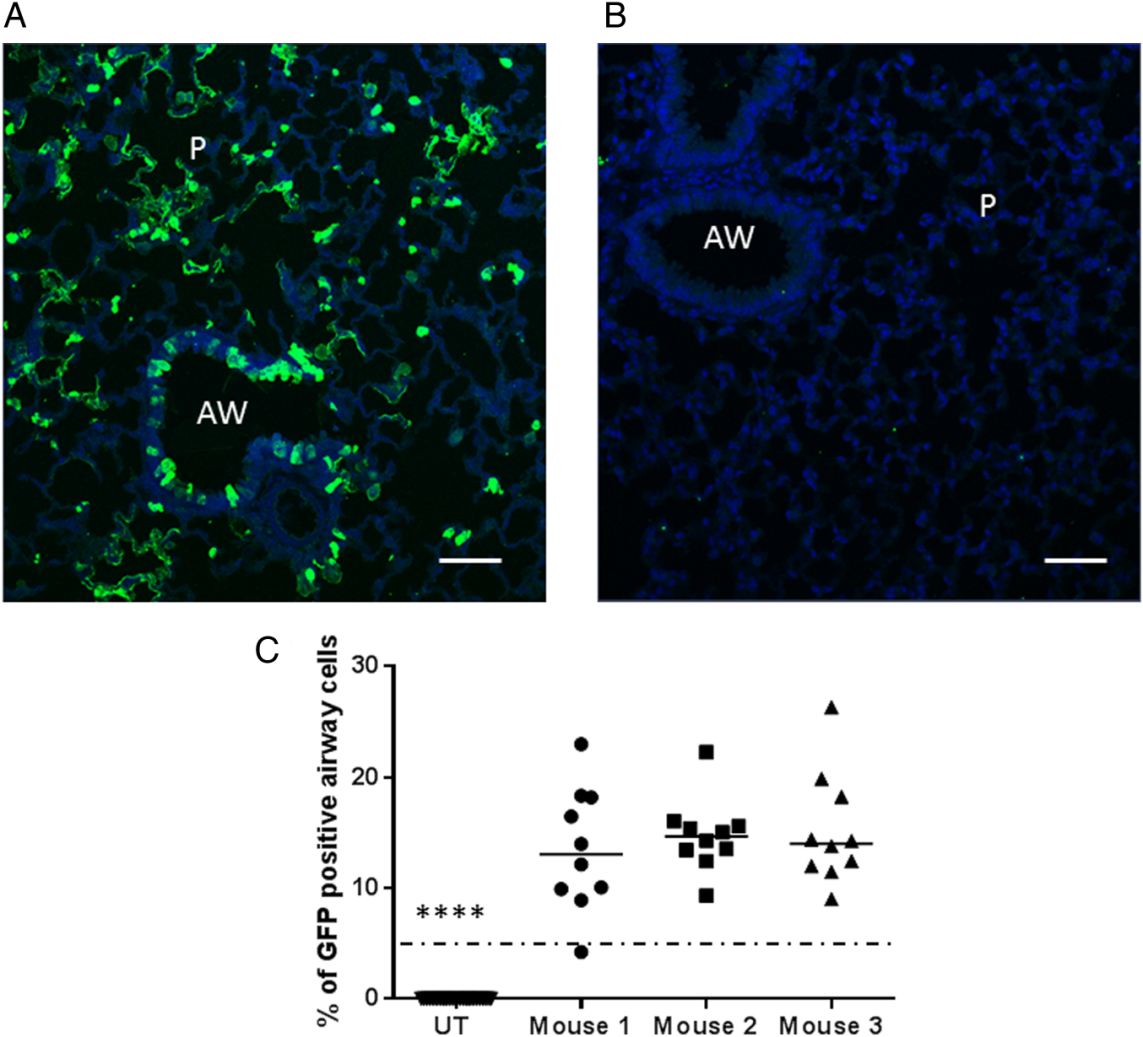
Gene expression in relevant airway epithelial cells. Mice were transduced with rSIV.F/HN-hCEF-enhanced green fluorescent protein (EGFP) (8E8 TU/mouse) or remained untreated (UT) (n=3/group). Seven days after transduction, mice were culled and the lungs processed for quantification of airway cells expressing EGFP by immunohistochemistry. (A) Representative image of a lentivirus transduced mouse. (B) Representative image of an UT mouse. Scale bar=50 µm. AW, airway; P, parenchyma. (C) Quantification of EGFP in mouse airways. Each dot represents a randomly selected airway (n=10/mouse). For convenience, the data from the UT control mice were pooled. The horizontal bar shows the median. The dotted horizontal line represents the consensus therapeutic threshold of 5% airway cells. ****p<0.0001 comparing all treated mice with controls (ANOVA followed by Dunnett's multiple comparison test). GFP, green fluorescent protein.

Murine lungs were transduced with rSIV.F/HN-hCEF carrying the enhanced green fluorescent protein (EGFP) cDNA and gene expression quantified histologically. Representative sections of airway and alveolar regions are shown in figure 2A, B. Approximately 15% of the target airway epithelial cells throughout the lung expressed EGFP (figure 2C).

**Figure 3:**
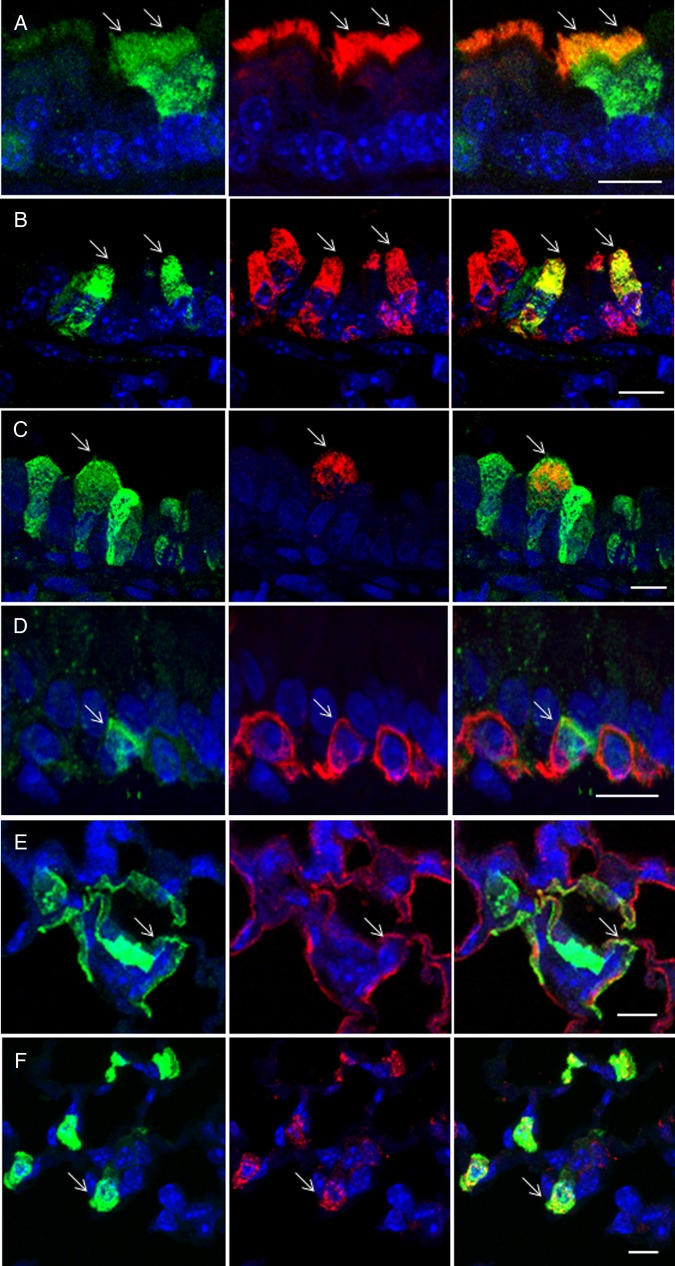
Characterisation of transduced cells by immunohistochemistry. Mice were transduced with rSIV.F/HN-hCEF-enhanced green fluorescent protein (EGFP) (8E8 TU/mouse) or remained untreated (n=3/group). Seven days after transduction, mice were culled and the lungs processed for characterisation of EGFP-expressing cells by immunohistochemistry. Tissue sections were double-stained with anti-EGFP and cell-type-specific antibodies and DAPI to visualise nuclei (blue). The left panel shows EGFP-expressing cells in green, the middle panel shows cell-type-specific staining in red and the right panel shows a merged image. Arrows highlight double-labelled cells. The merged images do not in all cases show a yellow/orange signal when green and red signals are overlaid because the proteins stained are localised to different cellular compartments, for example, in type 1 pneumocytes, the EGFP is present in the cytoplasm, whereas podoplanin is a membrane protein. Scale bar=10 µm. (A) Anti-β tubulin antibody identifies ciliated airway epithelial cells, (B) anti-uteroglobin antibody identifies club cells in the airways, (C) anti-mucin 5AC antibody identifies goblet cells in the airways, (D) anti-cytokeratin 5 antibody identifies basal cells in the airways, (E) anti-podoplanin antibody identifies type 1 pneumocytes and (F) anti-surfactant protein C antibody identifies type 2 pneumocytes.

To further characterise the range of cells that rSIV.F/HN-hCEF-EGFP transduced, double labelling using a range of cell-type-specific antibodies was performed (see online supplementary table S2). Figure 3 shows that the vector was also able to transduce goblet and club cells, as well as type I and II pneumocytes and on rare occasions basal cells. We could not detect EGFP expression in pulmonary macrophages.

### rSIV.F/HN shows a similar acute toxicology profile to liposome transfection

We have previously shown that F/HN-pseudotyped lentiviral vector administration to murine lung does not cause chronic toxicity.^4^ Here, we show that the survival kinetics of mice treated with various vectors manufactured using regulator-compliant, animal-product-free, production methods did not differ from vehicle-treated littermates and the animals remained healthy on gross observation (see online supplementary figure S1).

**Figure 4:**
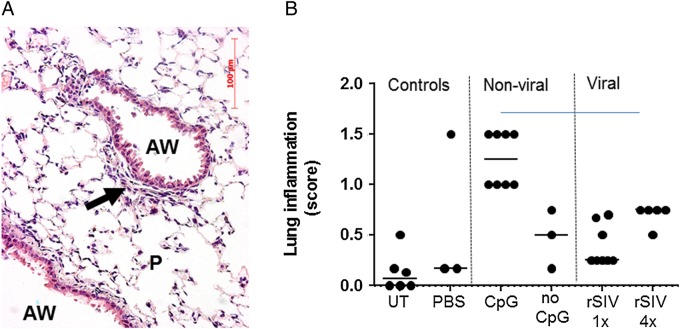
Assessment of acute pulmonary toxicity after lentivirus transduction. Mice were transduced with one or four doses (1E8 TU/dose at monthly intervals, n=5/group) of rSIV.F/HN-cytomegalovirus vectors carrying luciferase or enhanced green fluorescent protein reporter genes and histological analysis was performed 24 hours after the last dose. Control groups included UT and D-PBS-treated mice and mice treated with conventional (CpG containing) luciferase plasmid DNA/GL67A complexes or CpG-free CFTR plasmid pGM169/GL67A (n=3–5 mice/group). (A) Representative image of a lentivirus-treated mouse. AW, airway, P, parenchyma, arrow indicates mild cellular infiltrate, (B) semiquantitative scoring of lung inflammation. UT, untreated, one dose of rSIV (rSIV1x) and four monthly doses of rSIV (rSIV4x). Each symbol represents an individual mouse. The horizontal bar indicates the group median.

We next assessed the acute toxicity 24 hours after a single administration, as well as 24 hours after the final of four monthly doses of rSIV-F/HN. Mild cellular infiltrates were observed in all groups treated with lentivirus (representative image shown in figure 4A). However, these responses (figure 4B) were of similar magnitude to those produced by the non-viral formulation, which was recently used in a Phase IIb multidose trial and did not result in any significant toxicity in patients with CF^14^ and were not significantly different to changes seen in control animals.

### Insertion site analysis

Integration site frequency analyses calculated by GREAT using regions defined as integration site ±10 kb and integration site ±100 kb showed that 73% and 70%, respectively, of each were between 5 and 500 kb from transcription start sites (TSS) (see online supplementary figure S2 and the Results section in the online supplementary data). Although there are insufficient integration sites to draw definitive conclusions, an exploratory ontological survey revealed no preference for integration near oncogenic loci (data not shown).

### Effects of acquired immunity and pre-existing immunity

In the context of viral gene transfer, acquired immunity and pre-existing immunity to the vector are important considerations. We, and others, have previously shown that in contrast to other viral vectors, lentiviruses can be repeatedly administered at doses that are in a likely therapeutic range.^3^ ^4^ ^15^

### Acquired immunity—induction of rSIV.F/HN neutralising antibodies?

We first confirmed that the pharmacopoeia-compliant vector configuration and serum-free production methods did not affect the efficacy of repeat administration (three doses at monthly intervals) (Virus Production paper; in preparation). As part of these experiments, we also quantified neutralising antibodies using an in vitro transduction inhibition assay and showed that antibodies neutralising rSIV.F/HN, but not a vector pseudotyped with the vesicular stomatitis virus G glycoprotein (r.SIV.VSV-G, negative control) neutralising antibodies were detectable in serum of mice 28 days after a single dose of rSIV.F/HN (see online supplementary figure S3).

### Pre-existing immunity—passive immunisation of mice with human Ig

The key F and HN proteins on rSIV.F/HN are derived from SeV which has high sequence homology with human parainfluenza virus 1 (hPIV1).^16^ ^17^ Approximately 70–90% of people have been infected with hPIV1 and produce anti-hPIV1 antibodies. We, therefore, assessed whether the presence of anti-hPIV1 antibodies altered transduction efficiency and toxicity in mice. We first incubated rSIV.F/HN with either purified IgG or IgA anti-hPIV antibodies; both inhibited the transduction of rSIV./F/HN in vitro (see online supplementary figure S4).

To assess the in vivo significance of these findings, mice were then treated with human Igs (IVIg) intraperitoneally or topically to the lung by nasal sniffing. The doses achieved, and indeed exceeded, antibody titres measured in human broncho alveolar lavage fluid (BALF) and serum (see online supplementary figure S5). Unsurprisingly, intraperitoneal (IP) administration of IVIg leads to higher antibody levels in serum, whereas intratracheal administration leads to higher titres in BALF.

**Figure 5:**
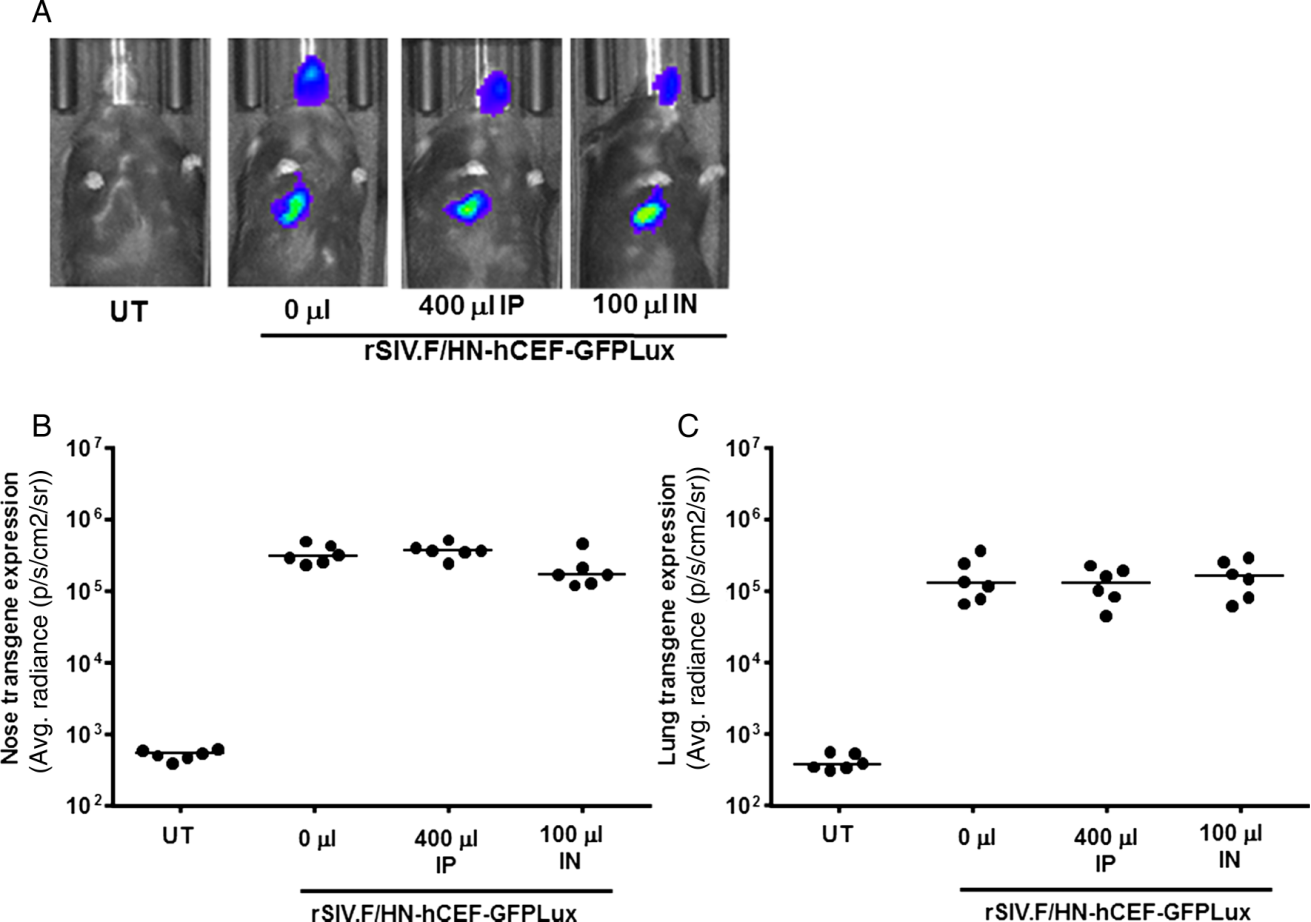
Transduction efficiency in mouse lung and nose in the presence of anti-human human parainfluenza virus 1 (hPIV1) antibodies. Mice were treated with human Ig (IVIg) intraperitoneally (IP, 400 μL) or by nasal instillation (IN, 100 μL). Controls did not receive IVIg (n=6/group). Twenty-four hours after passive immunisation, mice were transduced with rSIV.F/HN-hCEF-EGFPLux (1E8 TU/mouse). Control mice remained untreated (UT). Luciferase expression was quantified in lung and nose using bioluminescent imaging 24 hours after virus transduction. (A) Representative images for each cohort of mice, (B) luciferase expression nose and (C) lung. Each symbol represents one animal. The horizontal bar indicates the group median. Two independent experiments were performed (n=6/group/experiment) and a representative figure is shown. EGFP, enhanced green fluorescent protein; GFP, green fluorescent protein.

We next treated mice with IVIg IP and intranasally (IN) as described above, followed 24 hours later by rSIV.F/HN-hCEF-EGFPLux. There was no significant reduction in gene expression in the nose or lung 7 days (figure 5) or 28 days after transduction (data not shown).

We also assessed the potential toxicity arising from the interaction of rSIV.F/HN with the preformed hPIV1 antibodies. Gross observation showed that transduced mice pretreated with IVIg were indistinguishable from non-IVIg-treated mice. In addition, we monitored temperature in the acute phase immediately post virus administration and did not observe differences in any of the groups (see online supplementary figure S6A). Further, animal and lung weights did not differ in any of the groups (see online supplementary figures S6B, C).

### Pre-existing immunity–SeV-induced anti-F and HN antibodies

To further assess the effect of pre-existing immunity to the key F and HN epitopes, mice were challenged with SeV prior to transduction with rSIV.F/HN. Mice were transduced with two doses (1 month apart) of transmission-incompetent SeV^18^ ^19^ (1E6 or 1E7 TU/mouse/dose) followed by one dose of rSIV.F/HN-hCEF-EGFPLux. We confirmed that high levels (p<0.001) of anti-SeV antibodies were generated in BALF and serum after SeV transduction (see online supplementary figure S7E); transduction with rSIV.F/HN also increased (p<0.001) anti-SeV antibodies in serum. There was no difference in any of the groups (see online supplementary figure S7A–D) with respect to weight, food and water consumption or body temperature over time.

### Pre-existing immunity—neutralising activity of anti-hPIAV1 in human serum

We next quantified endogenous anti-hPIV1 IgG levels in serum from adults and children to enable IgG positive and negative samples to be selected for the in vitro transduction inhibition assay (see online supplementary figure S8A). rSIV.F/HN transduction was inhibited by anti-hPIAV1 IgG positive and negative samples (see online supplementary figure S8B–E). In contrast, and similar to the in vitro data obtained using murine, rSIV.VSV-G-mediated transduction was significantly (p<0.01) less affected confirming epitope specificity (see online supplementary figure S8F).

### Pre-existing immunity—neutralising activity of anti-hPIAV1 in epithelial lining fluid

To address whether these endogenous anti-hPIV1 antibodies in epithelial lining fluid (ELF) may inhibit rSIV.F/HN transduction in vivo, we first quantified anti-hPIV1 IgG and IgA antibodies in BALF from children and adults'. Approximately 5% of children and 35% of adult samples were positive for anti-hPIV1 IgG, while 47% of children's and 44% of adults' samples were positive for anti-hPIV1 IgA (see online supplementary figure E9A). We next grouped subjects into: IgG+/IgA+(n=4), IgG+/IgA− (n=5), IgG−/IgA+ (n=15) and IgG−/IgA− (n=17) and undertook in vitro transduction inhibition assays on 31 out of the 41 samples. The average % inhibition was 36±17%, 22±13%, 10±4% and 12±4% for the four groups, respectively. Subsequently, antibody positive samples (irrespective of type) were pooled and compared with antibody negative samples. There was no significant difference between the groups, suggesting that the low levels of inhibition seen were unlikely to be anti-hPIV1 antibody specific (see online supplementary figure S9B). As a control, we also assessed transduction inhibition of a VSV-G-pseudotyped control virus in a subset of samples, which was not different from rSIV.F/HN, suggesting that the modest inhibition is unrelated to anti-hPIV1 antibodies.

### CFTR expression and function after rSIV.F/HN-hCEF-CFTR transduction

**Figure 6:**
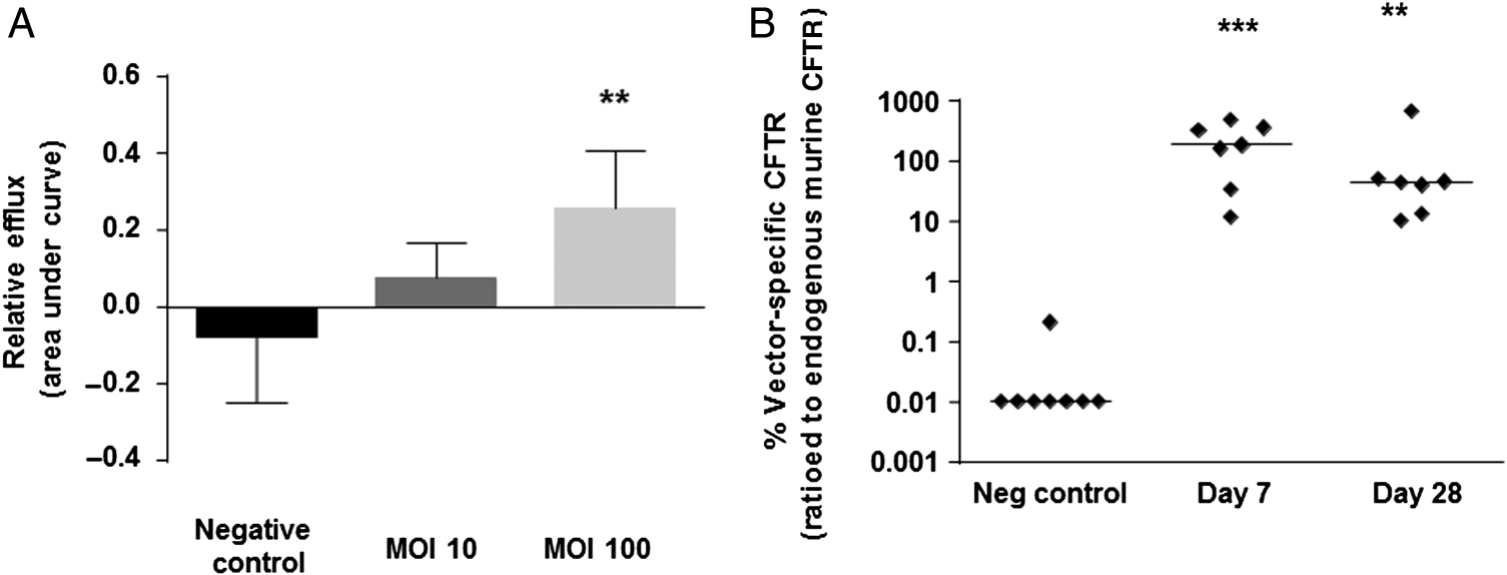
Confirmation of CFTR expression and function. (A) HEK293T cells were transfected with rSIV.F/HN-hCEF-CFTR or an irrelevant control virus (negative control) at MOIs of 10 and 100. The iodide efflux assay was performed 2 days after transduction. Data are presented as mean±SEM. **p<0.05 compared with negative control (ANOVA followed by Dunnett's multiple comparison test). (B) Cystic fibrosis knockout mice were transduced with rSIV.F/HN-hCEF-CFTR (1.6E8 TU/mouse) by nasal instillation. Negative controls were treated with PBS (n=7–8/group). Mice were culled 7 and 28 days after vector administration and vector-specific mRNA was quantified in the lungs. Each symbol represents one animal. The horizontal bar shows the group median. The dotted line indicates the detection limit of the assay. ***p<0.005 and **p<0.01 compared with the negative control (Kruskal-Wallis followed by Dunn's multiple comparison test).

We have previously shown that F/HN-SIV expressing CFTR under the CMV promoter generates CFTR chloride channels as assessed by the iodide efflux assay in vitro*.*^3^ Here, we first confirmed that the lead candidate rSIV.F/HN-hCEF carrying a codon-optimised and cytosine guanine dinucleotide (CG) nucleotide-depleted CFTR cDNA (soCFTR2) also generated cyclic Adenosine Monophosphate (cAMP)-dependent CFTR chloride channels in this assay (figure 6A).

We next transduced the nasal epithelium of CF knockout mice with rSIV.F/HN-hCEF-CFTR and detected significant levels of vector-specific mRNA at both 7 (p<0.005) and 28 (p<0.01) days (figure 6B). We also transduced CF knockout mice with rSIV.F/HN-hCEF-CFTR (5E7 TU/mouse, n=10) and assessed nasal potential difference at time points ranging from 7 to 90 days post transduction, but were unable to document correction of the chloride transport defect at this titre (data not shown, see online supplementary material for further discussion).

**Figure 7:**
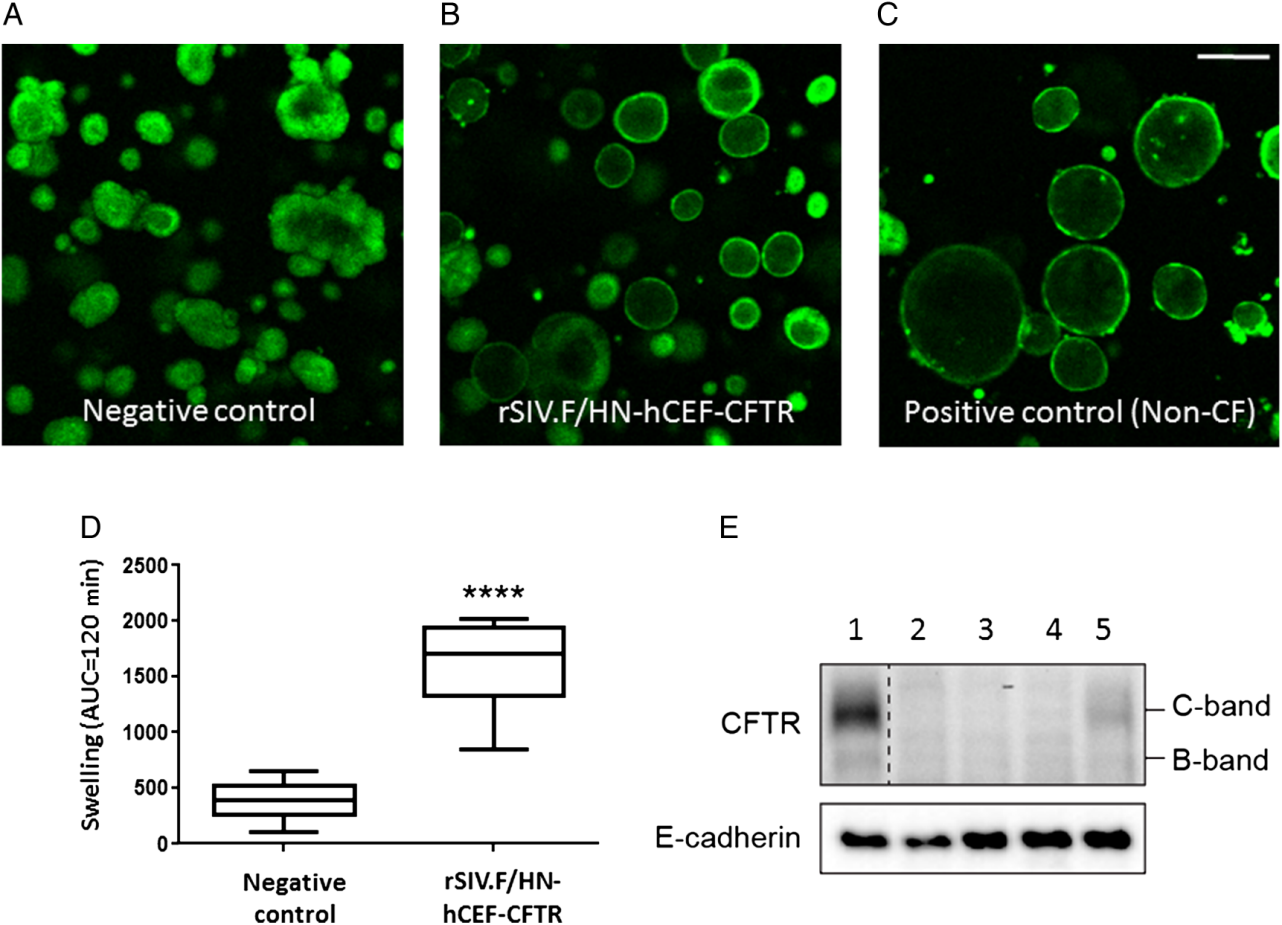
Functional confirmation of CFTR production in cystic fibrosis (CF) intestinal organoids. (A–D) CF intestinal organoids carrying two class I mutations (E60X/4015delATTT) were transduced with rSIV.F/HN-hCEF-CFTR or an irrelevant control virus (negative control). The doses in experiment 1 ranged from 0.45 to 3.6E7 TU/well and in experiment 2 from 0.06 to 0.45E7 TU/well (n=4/dose/experiment). Doses greater than 1.8E7 TU/well resulted in cell toxicity and reduced chloride transport (as measured by reduced organoid swelling upon forskolin addition) (data not shown). Analysis of chloride transport in the CF organoids 4 days after transduction therefore focused on samples treated with 0.23–0.9E7 TU/well (n=15–16 wells/group in two independent experiments). Four days post-transduction, organoid swelling upon addition of forskolin was assessed (measured as area under curve (AUC) over 120 min, baseline set at t=0). Representative organoid images are shown. Data are presented as mean±SEM. ****p<0.0001 compared with negative control (non-paired Student's t-test). (E) At the end of the experiment, organoids were harvested for protein extraction and western blot analysis. Lane 1: non-CF organoids transduced with negative control virus, Lanes 2–4: CF organoid untransduced or transduced with a negative control virus and Lane 5: CF organoids transduced with rSIV.F/HN-hCEF-CFTR.

Forskolin-induced swelling of human intestinal organoids has recently been shown to be an accurate readout for CFTR channel activity.^20^ We first assessed whether rSIV.F/HN-hCEF carrying a secreted GLux reporter gene could transduce non-CF organoids. High levels of GLux expression were detected in all treated samples (see online supplementary figure S10). We next transduced CF organoids with rSIV.F/HN-hCEF-CFTR. We observed that organoid swelling increased significantly (p<0.0001) in rSIV.F/HN-hCEF-CFTR-treated cultures compared with negative controls (figure 7A–D). These functional data were supported by western blot detection of CFTR protein in CF organoids (figure 7E). In conclusion, the data indicate that lentiviral delivery of CFTR in CF intestinal organoids partially restores their CF characteristics.

### Vector stability in clinically relevant delivery devices

Vector stability was assessed in a range of delivery devices suitable for administration of lentivirus to the nose or a restricted region in the lungs of patients with CF. Ultimately, we anticipate the vector to be delivered via an aerosol-generating nebuliser to the whole lung. However, first-in-man safety and proof-of-concept studies may focus on local and directed delivery of the vector to the nasal and airway epithelium. We therefore assessed vector stability in two catheters and in a nasal spray bottle and showed that passage through these devices did not reduce transduction efficiency (see online supplementary figure S11). Thus, clinically relevant delivery devices suitable for administration to the nose as well as for regional lung delivery have been identified.

## Discussion

In addition to the use of SIN vectors, the regulation of gene expression by internal promoter/enhancer elements, rather than by the viral LTRs appears to have significantly improved biosafety of the vectors^21^ and has highlighted the importance of optimisation of the expression cassettes in improving efficacy. We compared the strong CMV promoter/enhancer (recently used in Parkinson's disease trials^22^), the human EF1α short promoter (a commonly used ubiquitous eukaryotic promoter capable of persistent transgene expression in the lung following non-viral gene transfer)^23^ and a CpG-free hybrid promoter (hCEF) consisting of the EF1α promoter and the human CMV enhancer, which we initially developed for, and have recently used in, our Phase IIb non-viral CF gene therapy trial.^14^ ^24^ The hCEF regulatory element led to the highest levels of gene expression in the murine lung and nose in vivo as well as in human ALI cultures. However, we noted good consistency between murine lung and nose as well as the human ALI models and these data may help inform future strategies for screening promoter/enhancer elements for lung delivery. As part of our screening strategy, we also compared standard IC and ID vectors. In our models, the profile of low transient expression from the ID vector configurations did not support further assessment.

It is likely that the hCEF regulatory element leads to higher than physiological expression of CFTR in transduced cells. Although it has been shown that CFTR overexpression can affect cell proliferation rates in vitro, transgenic mice overexpressing CFTR showed no adverse effects.^25^ In addition, we have not seen any evidence of CFTR-related toxicity in our recent multidose non-viral gene therapy trial, which also used the hCEF regulatory element.^14^

Using our lead candidate configuration, approximately 15% of relevant target epithelial were transduced at the titres we were able to generate for these studies. This compares favourably with a broadly held view that between 5% and 25% of cells may require correction to provide a meaningful level of clinical correction. The titre of 8E8 TU of rSIV-F/HN-hCEF-EGFP used to generate these data will form the basis for estimating suitable dose ranges for the first-in-man clinical trial.

In addition to ciliated airway epithelial cells, a range of other cell types, including goblet and club cells, as well as type I and II pneumocytes were transduced. Consistent with our previous data in the nasal epithelium,^3^ we confirmed that basal cells, likely progenitor cells located in the subepithelial layer, were only infrequently transduced. We did not see any evidence for transduction of pulmonary macrophages, the preferential cell type transduced by VSV-G-pseudotyped lentivirus when applied to the mouse lung by bolus administration and without the addition of a tight junction opener.^26^ The broad transduction range of rSIV.F/HN is not surprising considering that recombinant SeV similarly transduces a wide range of cell types.^27^ In the human lung, the range of transduced cell types will likely depend on the delivery method. For example, aerosols with droplet sizes of 3–5 μm, which are most suitable for airway delivery, will, for example, not lead to efficient vector deposition in the alveoli.

Consistent with our previous findings, transduction with pharmacopoeia-compliant vectors did not cause chronic or excessive acute toxicity. The mild neutrophilic infiltrates observed in mouse lung were of similar magnitude to those produced by our non-viral formulation pGM169/GL67A, which has already been assessed in a multidose Phase IIb trial.^14^

Oncogenesis through viral genome integration is intrinsically less likely in terminally differentiated cells of the airway epithelium than it is in rapidly dividing cells of the haematopoetic lineage. We infer from this preliminary study that there is no obvious bias in the distribution of integration site at the chromosomal level; the integration pattern in relation to TSS is similar to that reported for an SIV-based vector in primary haematopoietic stem cells by Nienhuis *et al.*^28^ Given the terminally differentiated nature of the airway epithelium, it is notable that the findings are broadly consistent with lentiviral integration site distributions in the eye and brain,^29^ ^30^ suggesting that the genotoxicity risk of rSIV.F/HN vectors in the airways is comparably low.

Acquired and pre-existing immune responses have affected the use of adenoviral-associated and adeno-associated virus vectors for airway gene transfer.^31^ ^32^ We and others have previously shown^4^ ^15^ that lentiviral vectors can be repeatedly administered. We also show that repeated administration is feasible despite detection of anti-rSIV.F/HN neutralising antibodies in serum. It is difficult to make quantitative comparisons between antibody levels obtained in this and other studies due to the variability of the in vitro transduction inhibition assays used. However, Sinn *et al*^15^ reported that induction of humoral immune responses after lentivirus pulmonary gene transfer was significantly lower than after adenovirus-mediated gene transfer to the lung. It is currently unclear whether repeated administration of lentiviral vectors is feasible because of (a) low immunogenicity, (b) rapid cell entry thereby avoiding contact with neutralising antibodies or (c) due to other reasons. It is also unclear how well animal models will predict responses in man. In the context of Adeno-associated virus (AAV)-mediated liver transduction, it has become clear that murine models did not predict immune responses in man.^33^

To assess the effects of pre-existing human antibodies that may cross-react with the F and HN proteins and affect efficacy and toxicity, we pretreated mice with human Igs (IVIg) which contain anti-hPIAV1 IgG. IVIg was administered either IP or IN and the dosing strategy achieved hPIAV1 antibody titres in ranges representative of titres in human serum and BALF. The presence of pre-existing hPIV antibodies did not alter the transduction efficiency or safety in mice. In contrast, it has previously been shown that IVIg preadministration (12 mg/mouse) drastically reduces AAV-mediated gene transfer to the liver,^34^ thereby validating the use of human antibodies in murine models.

The cause of death of a participant in an early adenovirus clinical trial in 1999 has been widely debated.^35^ One suggestion is that the presence of high level, pre-existing, anti-adenovirus antibodies may have led to complement activation after vector administration initiating a severe immune reaction.^36^ To assess whether pre-existing antibodies to F and HN proteins alter rSIV.F/HN toxicity, we pretreated mice with two doses of SeV prior to lentivirus transduction and did not observe enhanced toxicity compared with controls.

The majority of humoral response against hPIV is mediated by IgG and IgA antibodies.^37^ IgA is the predominant Ig in the upper respiratory tract where it is locally synthesised by plasma cells in the lamina propria. IgG is the main Ig isotype in blood and in the lower respiratory tract. We, therefore, next assessed transduction inhibition of rSIV.F/HN in human serum samples that were either positive or negative for anti-hPIV1 IgG and showed that transduction inhibition occurred in both hPIV1 ELISA positive and negative samples, whereas the transduction with rSIV.VSV-G was not inhibited. The reasons for the inhibition in hPIV1 negative samples may relate to sensitivity of the ELISA assay or cross-reactivity with antibodies directed against other hPIV serotypes, which the hPIV-specific ELISA would not have detected, but could affect the transduction efficiency. We then assessed transduction inhibition in BALF which contains IgG (derived from blood) and IgA (produced locally in the lung). We analysed anti-hPIAV1 IgG/IgA positive and negative samples and did not detect evidence for rSIV.F/HN-specific inhibition. We have previously determined that lavage fluid represents an approximately 40-fold dilution of the ELF using a standard urea assay (data not shown) and this may affect interpretation of the results. However, Moss *et al*^38^ detected anti-AAV2 neutralising antibodies in BALF of patients with CF treated with AAV2 and Bastian and Bewig^39^ showed that anti-adenovirus neutralising antibodies can be detected in human BALF, thus supporting the notion that vector neutralising antibodies can be detected in BALF despite the ELF dilution factor.

We have previously shown that F/HN-SIV vector expressing CFTR under the control of the CMV promoter generated cAMP-dependent ion transport in an in vitro iodide efflux assay^3^ and here confirmed these data for the pharmacopoeia-compliant rSIV.F/HN vector carrying the soCFTR2 cDNA. In addition, we demonstrated rSIV.F/HN transduction and CFTR function in a recently developed intestinal organoid model.^20^

We also showed that rSIV.F/HN-hCEF-CFTR transduces the nasal epithelium of CF knockout mice efficiently (∼100% vector-specific mRNA compared with endogenous murine Cftr mRNA).

CF mice do not acquire spontaneous airway infections or develop CF lung disease, but the nasal epithelium shows the characteristic CF chloride and sodium transport defects.^40^ To further assess CFTR function, we attempted to correct ion transport in the CF mouse nasal epithelium, but were unable to do so. For these experiments, we used a dose of 5E7 TU/mouse (maximum feasible dose based on vector availability). We cannot exclude the possibility that this titre may have been subtherapeutic. However, the relevance of measurement of CFTR function in the murine nose (via in vivo potential difference) has been called into question by Ostrowski *et al*^41^ who showed that expression of human CFTR under the transcriptional control of a cilia-specific promoter did not correct ion transport in CF knockout mice. In addition, Grubb *et al*^42^ have suggested that the olfactory, rather than the respiratory, nasal epithelium mainly contributes to the ion transport defect in CF mice. Considering these data, we do not expect an increase in vector dose to alter chloride secretion because we have previously shown that our vector does not efficiently transduce olfactory epithelial cells.^3^ We have also shown that the CF mouse is of limited value as a stepping stone to human gene therapy trials. Although GL67A-mediated *CFTR* gene transfer partially corrected chloride transport in the human lung and nose, reduced bacterial adherence to epithelial cells and decreased interleukin (IL)-8 and neutrophils in CF sputum,^43^ we were unable to correct a panel of CFTR-specific endpoint assays in the murine nose, including ion transport, periciliary liquid height and ex vivo bacterial adherence.^44^ Our data are also consistent with an earlier study by Jiang *et al*,^45^ who showed that GL67A-mediated gene transfer did not lead to correction of the ion transport defect in CF mice and our own report of successful correction of chloride transport in the human, but not in the murine, nose after transfection with DC-Chol/DOPE.^46^ ^47^ Taken together, these data suggest that the CF knockout mouse may not be a representative model in which to assess gene transfer efficiency to human airway epithelial cells and that correction of ion transport in mice should not be used as a go-no-go decision point for progression into clinical trial. We have also considered the two CF pig models that have been developed, but these animals currently die shortly after birth due to intestinal disease and, therefore, are (a) not available in large enough numbers to conduct meaningful studies and (b) not compatible with the time course of lentivirus integration and gene expression.

In preparation for a first-in-man trial, which will involve regional delivery of vector to the airways, we assessed vector stability in a range of delivery devices suitable for focal delivery. The virus was stable in these ‘single-pass’ delivery devices. We will conduct a single-dose, double-blinded, dose-escalating Phase I/IIa safety and efficacy clinical study. A total of 24 adult subjects will be recruited into four groups receiving 1E8, 5E8 and 2.5E9 TU of rSIV.F/HN-hCEF-CFTR or placebo. Dosage levels were determined principally by considering the titre necessary to demonstrate gene expression in the mouse nose, the target for therapeutic expression in humans (5% cells transduced) and the interspecies scaling factor. The trial will not be designed to detect clinical efficacy, but will focus on assessing safety and the time course of CFTR expression and function.

In summary, in combination with the parallel development of scalable good manufacturing practices (GMP)-compliant vector production methods, we suggest that the mouse and ex vivo human data presented here support the progression of rSIV.F/HN into a first-in-man clinical study for CF scheduled to start in 2017. In addition, the unique feature of this vector platform also opens opportunities for other lung and systemic diseases.

## References

[1] Kobayashi M, Iida A, Ueda Y, et al Pseudotyped lentivirus vectors derived from simian immunodeficiency virus SIVagm with envelope glycoproteins from paramyxovirus. J Virol 2003;77:2607–14. 10.1128/JVI.77.4.2607-2614.2003 12551999 PMC141089

[2] Griesenbach U, McLachlan G, Owaki T, et al Validation of recombinant Sendai virus in a non-natural host model. Gene Ther 2011;18:182–8. 10.1038/gt.2010.131 20962870

[3] Mitomo K, Griesenbach U, Inoue M, et al Toward gene therapy for cystic fibrosis using a lentivirus pseudotyped with Sendai virus envelopes. Mol Ther 2010;18:1173–82. 10.1038/mt.2010.13 20332767 PMC2889732

[4] Griesenbach U, Inoue M, Meng C, et al Assessment of F/HN-pseudotyped lentivirus as a clinically relevant vector for lung gene therapy. Am J Respir Crit Care Med 2012;186:846–56. 10.1164/rccm.201206-1056OC 22955314 PMC3530223

[5] Cmielewski P, Anson DS, Parsons DW Lysophosphatidylcholine as an adjuvant for lentiviral vector mediated gene transfer to airway epithelium: effect of acyl chain length. Respir Res 2010;11:84 10.1186/1465-9921-11-84 20569421 PMC2905357

[6] Sinn PL, Cooney AL, Oakland M, et al Lentiviral vector gene transfer to porcine airways. Mol Ther Nucleic Acids 2012;1:e56 10.1038/mtna.2012.47 23187455 PMC3511674

[7] Hacein-Bey Abina S, Gaspar HB, Blondeau J, et al Outcomes following gene therapy in patients with severe Wiskott-Aldrich syndrome. JAMA 2015;313:1550–63. 10.1001/jama.2015.3253 25898053 PMC4942841

[8] Fischer A, Hacein-Bey-Abina S, Cavazzana-Calvo M Gene therapy of primary T cell immunodeficiencies. Gene 2013;525:170–3. 10.1016/j.gene.2013.03.092 23583799

[9] Boztug K, Schmidt M, Schwarzer A, et al Stem-cell gene therapy for the Wiskott-Aldrich syndrome. N Engl J Med 2010;363:1918–27. 10.1056/NEJMoa1003548 21067383 PMC3064520

[10] Aiuti A, Biasco L, Scaramuzza S, et al Lentiviral hematopoietic stem cell gene therapy in patients with wiskott-aldrich syndrome. Science 2013;341: 1233151 10.1126/science.1233151 23845947 PMC4375961

[11] Biffi A, Montini E, Lorioli L, et al Lentiviral hematopoietic stem cell gene therapy benefits metachromatic leukodystrophy. Science 2013;341:1233158 10.1126/science.1233158 23845948

[12] Palfi S, Gurruchaga JM, Ralph GS, et al Long-term safety and tolerability of ProSavin, a lentiviral vector-based gene therapy for Parkinson's disease: a dose escalation, open-label, phase 1/2 trial. Lancet 2014;383:1138–46. 10.1016/S0140-6736(13)61939-X 24412048

[13] Yáñez-Muñoz RJ, Balaggan KS, MacNeil A, et al Effective gene therapy with nonintegrating lentiviral vectors. Nat Med 2006;12:348–53. 10.1038/nm1365 16491086

[14] Alton EW, Armstrong DK, Ashby D, et al Repeated nebulisation of non-viral CFTR gene therapy in patients with cystic fibrosis: a randomised, double-blind, placebo-controlled, phase 2b trial. Lancet Respir Med 2015;3:684–91. 10.1016/S2213-2600(15)00245-3 26149841 PMC4673100

[15] Sinn PL, Arias AC, Brogden KA, et al Lentivirus vector can be readministered to nasal epithelia without blocking immune responses. J Virol 2008;82:10684–92. 10.1128/JVI.00227-08 18768988 PMC2573216

[16] Gorman WL, Gill DS, Scroggs RA, et al The hemagglutinin-neuraminidase glycoproteins of human parainfluenza virus type 1 and Sendai virus have high structure-function similarity with limited antigenic cross-reactivity. Virology 1990;175:211–21. 10.1016/0042-6822(90)90201-2 1689918

[17] Lamb RA, Kolakofsky D Paramyxoviridae: the viruses and their replication. In: Fields BN, Knipe DM, Howley PM Fields virology. 3rd edn. Philadelphia, PA: Lippincott-Raven Publishers, 1996:1177–204.

[18] Ferrari S, Griesenbach U, Iida A, et al Sendai virus-mediated CFTR gene transfer to the airway epithelium. Gene Ther 2007;14:1371–9. 10.1038/sj.gt.3302991 17597790

[19] Griesenbach U, Boyton RJ, Somerton L, et al Effect of tolerance induction to immunodominant T-cell epitopes of Sendai virus on gene expression following repeat administration to lung. Gene Ther 2006;13:449–56. 10.1038/sj.gt.3302677 16319950

[20] Dekkers JF, Wiegerinck CL, de Jonge HR, et al A functional CFTR assay using primary cystic fibrosis intestinal organoids. Nat Med 2013;19:939–45. 10.1038/nm.3201 23727931

[21] Zhang L, Thrasher AJ, Gaspar HB Current progress on gene therapy for primary immunodeficiencies. Gene Ther 2013;20:963–9. 10.1038/gt.2013.21 23719067

[22] Stewart HJ, Leroux-Carlucci MA, Sion CJ, et al Development of inducible EIAV-based lentiviral vector packaging and producer cell lines. Gene Ther 2009;16:805–14. 10.1038/gt.2009.20 19262613

[23] Gill DR, Smyth SE, Goddard CA, et al Increased persistence of lung gene expression using plasmids containing the ubiquitin C or elongation factor 1alpha promoter. Gene Ther 2001;8:1539–46. 10.1038/sj.gt.3301561 11704814

[24] Hyde SC, Pringle IA, Abdullah S, et al CpG-free plasmids confer reduced inflammation and sustained pulmonary gene expression. Nat Biotechnol 2008;26:549–51. 10.1038/nbt1399 18438402

[25] Farmen SL, Karp PH, Ng P, et al Gene transfer of CFTR to airway epithelia: low levels of expression are sufficient to correct Cl- transport and overexpression can generate basolateral CFTR. Am J Physiol Lung Cell Mol Physiol 2005;289:L1123–30. 10.1152/ajplung.00049.2005 16085675

[26] Wilson AA, Murphy GJ, Hamakawa H, et al Amelioration of emphysema in mice through lentiviral transduction of long-lived pulmonary alveolar macrophages. J Clin Invest 2010;120:379–89. 10.1172/JCI36666 20038801 PMC2798672

[27] Griesenbach U, Inoue M, Hasegawa M, et al Sendai virus for gene therapy and vaccination. Curr Opin Mol Ther 2005;7:346–52. 16121700

[28] Nienhuis AW, Dunbar CE, Sorrentino BP Genotoxicity of retroviral integration in hematopoietic cells. Mol Ther 2006;13:1031–49. 10.1016/j.ymthe.2006.03.001 16624621

[29] Bartholomae CC, Arens A, Balaggan KS, et al Lentiviral vector integration profiles differ in rodent postmitotic tissues. Mol Ther 2011;19:703–10. 10.1038/mt.2011.19 21364536 PMC3070105

[30] Lattanzi A, Salvagno C, Maderna C, et al Therapeutic benefit of lentiviral-mediated neonatal intracerebral gene therapy in a mouse model of globoid cell leukodystrophy. Hum Mol Genet 2014;23:3250–68. 10.1093/hmg/ddu034 24463623 PMC4030779

[31] Yang Y, Li Q, Ertl HC, et al Cellular and humoral immune responses to viral antigens create barriers to lung-directed gene therapy with recombinant adenoviruses. J Virol 1995;69:2004–15. 7884845 10.1128/jvi.69.4.2004-2015.1995 PMC188865

[32] Moss RB, Milla C, Colombo J, et al Repeated aerosolized AAV-CFTR for treatment of cystic fibrosis: a randomized placebo-controlled phase 2B trial. Hum Gene Ther 2007;18:726–32. 10.1089/hum.2007.022 17685853

[33] Nichols TC, Whitford MH, Arruda VR, et al Translational data from adeno-associated virus-mediated gene therapy of hemophilia B in dogs. Hum Gene Ther Clin Dev 2015;26:5–14. 10.1089/humc.2014.153 25675273 PMC4442577

[34] Wilson JM, Limberis M The challenge of developing animal models of human gene therapy with AAV [abstract]. Mol Ther 2011;19:S1–S361.21638795

[35] Thomas CE, Ehrhardt A, Kay MA Progress and problems with the use of viral vectors for gene therapy. Nat Rev Genet 2003;4:346–58. 10.1038/nrg1066 12728277

[36] Cichon G, Boeckh-Herwig S, Schmidt HH, et al Complement activation by recombinant adenoviruses. Gene Ther 2001;8:1794–800. 10.1038/sj.gt.3301611 11803399 PMC7091591

[37] Henrickson KJ Parainfluenza viruses. Clin Microbiol Rev 2003;16:242–64. 10.1128/CMR.16.2.242-264.2003 12692097 PMC153148

[38] Moss RB, Rodman D, Spencer LT, et al Repeated adeno-associated virus serotype 2 aerosol-mediated cystic fibrosis transmembrane regulator gene transfer to the lungs of patients with cystic fibrosis: a multicenter, double-blind, placebo-controlled trial. Chest 2004;125:509–21. 10.1378/chest.125.2.509 14769732

[39] Bastian A, Bewig B Inhibition of adenovirus-mediated gene transfer by bronchoalveolar lavage fluid. Gene Ther 1999;6:637–42. 10.1038/sj.gt.3300854 10476223

[40] Griesenbach U, Smith SN, Farley R, et al Validation of nasal potential difference measurements in gut-corrected CF knockout mice. Am J Respir Cell Mol Biol 2008;39:490–6. 10.1165/rcmb.2007-0385OC 18458238

[41] Ostrowski LE, Yin W, Diggs PS, et al Expression of CFTR from a ciliated cell-specific promoter is ineffective at correcting nasal potential difference in CF mice. Gene Ther 2007;14:1492–501. 10.1038/sj.gt.3302994 17637798

[42] Grubb BR, Rogers TD, Boucher RC, et al Ion transport across CF and normal murine olfactory and ciliated epithelium. Am J Physiol, Cell Physiol 2009;296:C1301–9. 10.1152/ajpcell.00578.2008 19321738 PMC2692423

[43] Alton EW, Stern M, Farley R, et al Cationic lipid-mediated CFTR gene transfer to the lungs and nose of patients with cystic fibrosis: a double-blind placebo-controlled trial. Lancet 1999;353:947–54. 10.1016/S0140-6736(98)06532-5 10459902

[44] Griesenbach U, Sumner-Jones SG, Holder E, et al Limitations of the murine nose in the development of nonviral airway gene transfer. Am J Respir Cell Mol Biol 2010;43:46–54. 10.1165/rcmb.2009-0075OC 19648474

[45] Jiang C, O'Connor SP, Fang SL, et al Efficiency of cationic lipid-mediated transfection of polarized and differentiated airway epithelial cells in vitro and in vivo. Hum Gene Ther 1998;9:1531–42. 10.1089/hum.1998.9.11-1531 9694152

[46] Caplen NJ, Alton EW, Middleton PG, et al Liposome-mediated CFTR gene transfer to the nasal epithelium of patients with cystic fibrosis. Nat Med 1995;1:39–46. 10.1038/nm0195-39 7584951

[47] Alton EW, Middleton PG, Caplen NJ, et al Non-invasive liposome-mediated gene delivery can correct the ion transport defect in cystic fibrosis mutant mice. Nat Genet 1993;5:135–42. 10.1038/ng1093-135 7504552

[48] Zhang F, Frost AR, Blundell MP, et al A ubiquitous chromatin opening element (UCOE) confers resistance to DNA methylation-mediated silencing of lentiviral vectors. Mol Ther 2010;18:1640–9. 10.1038/mt.2010.132 20588258 PMC2956914

