# Supplementary material for: Preparation for a first-in-man lentivirus trial in patients with cystic fibrosis
Source: Thorax. 2016 Nov 16;72(2):137–47. doi: 10.1136/thoraxjnl-2016-208406 (PMC5284333; doi:10.1136/thoraxjnl-2016-208406)
Supplement: Supplementary data [file thoraxjnl-2016-208406supp.pdf]

Preparation for a first-in-man lentivirus trial in cystic fibrosis patients

Eric WFW. Alton, Jeffery M Beekman, A. Christopher Boyd, June Brand, Marianne S. Carlon, Mary M. Connolly, Mario Chan, Sinead Conlon, Heather E Davidson, Jane C. Davies, Lee A. Davies, Johanna F. Dekkers, Ann Doherty, Sabrina Gea-Sorli, Deborah R. Gill, Uta Griesenbach, Mamoru Hasegawa, Tracy E Higgins, Takashi Hironaka, Laura Hyndman, Gerry McLachlan, Makoto Inoue, Stephen C. Hyde, J. Alastair Innes, Toby M Maher, Caroline Moran, Cuixiang Meng, Michael C Paul-Smith, Ian A. Pringle, Kamila M Pytel, Andrea Rodriguez-Martinez, Alexander C Schmidt, Barbara J Stevenson, Stephanie G. Sumner-Jones, Richard Toshner, Shu Tsugumine, Marguerite W. Wasowicz, Jie Zhu

Materials and Methods

Generation of pharmacopoeia-compliant producer plasmid

Five producer plasmids were required to generate recombinant SIV vector. The sequences were as described(1), except that the ampicillin antibiotic-resistance gene was replaced with the CpG-free kanamycin antibiotic resistance gene from plasmid pGM169(2). For preparation of lentiviral vectors pseudotyped with Vesicular Stomatitis Virus G glycoprotein (VSV-G), the two plasmids expressing the F and HN proteins were replaced with one plasmid expressing VSV-G; full details of the construction and sequences of the pharmacopoeia-compliant producer plasmids will be published elsewhere (Virus Production paper; In Preparation).

In brief, multiple vector genome plasmids were constructed containing a variety of transgenes and transcription elements. DNA fragments encoding reporter transgenes such as Luciferase (lux), secreted Gaussia luciferase (GLux), Enhanced Green Fluorescent Protein (EGFP), and a fusion of EGFP and Luciferase (EGFPLux) were inserted into unique NheI and ApaI restriction sites in the vector genome plasmid. In addition, a vector genome plasmid expressing Cystic Fibrosis Transmembrane Conductance Regulator (CFTR) protein was constructed by insertion of the CpG-free NheI-ApaI DNA fragment encoding codon-optimised soCFTR2 from plasmid pGM169(2).

Promoter/enhancer sequences, including CMV(3), hCEF(2) and EF1α(3), were incorporated into the vector genome plasmid via BglII and NheI restriction sites, following removal of the intron, or synthetic intronic sequence. To generate integrase-defective (ID) vectors, a D64V point mutation in the integrase gene(4) was incorporated into the gag/pol packaging plasmid.

Vector production and titration

Production of recombinant SIV vector expressing a variety of transgenes was performed using the five-plasmid transient transfection method(1), except that 25K branched Polyethylenimine (PEI)(5) was used for transfection of producer plasmids into HEK293T producer cells grown in suspension culture in Freestyle media (Life Technologies, Paisley, UK). Virus supernatant was harvested at 72 hours post-transfection and purified essentially as described by Merten *et al*(6) except that Mustang QXT Anion Exchange membranes (Pall, Life Sciences, Portsmouth, UK) were used instead of DEAE columns (Virus Production paper; In Preparation). The virus was formulated in Freestyle medium aliquoted and stored at -80^{0}C.

The viral particle titre (VP/ml) was determined essentially as described by Mitomo *et al*(1), using Real-Time Quantitative PCR (Q-PCR) with primers spanning the WPRE sequence (Forward: TGGCGTGGTGTGCACTGT; Reverse: CCCGGAAAGGAGCTGACA; Probe: 6FAM-TTGCTGACGCAACCCCCACTGG-TAMRA). Virus RNA was prepared using QIAamp Viral RNA kit including carrier RNA (QIAGEN, Crawley, UK), followed by in-solution DNAse (Ambion, DNAfree) and quantified by one-step RT-qPCR using QuantiTect (QIAGEN) against a standard curve of RNA mimics containing the WPRE sequence (Virus Production paper; In preparation). This assay was also used to measure vector-specific RNA expression following transduction.

Functional titre, reported as transducing units per ml (TU/ml) was calculated following transduction of HEK293F cells with serial dilutions of viral vector and extraction of DNA using QIAamp blood DNA kit (QIAGEN) (Virus Production paper; In preparation). Viral DNA genomes were quantified by Q-PCR (same WPRE primers as above) against a standard curve of plasmid DNA containing the WPRE sequence, using TaqMan Universal Mastermix (Life Technologies), then normalised to total ng DNA using PicoGreen (LifeTechnologies) or Nanodrop ND2000 (Thermo Scientific, MA, USA). Titre was calculated from the slope of the

best-fit line on a plot of WPRE copies per well of cells against volume of virus per well of cells. This assay was also used to calculate Vector Copy Number (VCN) in transduced cells and tissues.

Vector Transduction:

All animal procedures were performed in accordance with the conditions and limitation of the UK Home Office Project and Personal licence regulations under the Animal Scientific Procedure Act (1986). Female C57BL/6N mice (6–8 wk old, Charles River Laboratories, UK) were used for most experiments. Male and female gut-corrected CF knockout mice (~6-12 weeks)(7) were used for assessment of CFTR expression and function. Mouse nose and lungs and air liquid interface (ALI) cultures were transduced with the vector as previously described(1) (see RESULTS and FIGURES for details about vector titers used) and transduction efficiency was quantified using bioluminescent imaging (BLI, IVIS, PerkinElmer, USA), luciferase expression in tissue homogenates as previously described(1) and immunohistochemistry (see below).

Integration site profiling

Genomic DNA was extracted (AllPrep, Life Technologies, Glasgow, UK) from transduced human air liquid interface (hALI) cultures and mouse nose and lung tissue samples. Viral copy number (VCN) was determined as described above. LAM-PCR was carried out according to Schmidt *et al*(8) with minor modifications. Two cocktails of enzymes, GC (HinPII, HpyCH4IV, HpaII, TaqI: NEB, UK) and AT (MseI, BfaI, CviQI: NEB, UK) were used to maximise flanking gDNA amplification. 25-150 ng of template gDNA was used for each LAM-PCR procedure. To increase the probability of IS retrieval, three separate linear amplification reactions of 300 cycles were pooled and then divided into three identical

triplicate aliquots. Products were size-fractionated after the nested PCR stage by using low melting point agarose gel extraction to remove DNA<100 bp. The length profile and band diversity of amplicons were visually assessed using a bioanalyser (Agilent Technologies, Stockport, UK) before proceeding. Purified PCR products were then prepared for Ion Torrent sequencing as recommended by the manufacturer (Life Technologies, Glasgow, UK) and sequenced. Sequencing reads were processed bioinformatically using a pipeline of custom Perl scripts to validate the input and remove superfluous sequences of the primers, linkers, remaining 32 bp of LTR and other vector sequences. The pipeline allows two levels of quality control based on degree of homology to the reference genomes termed *high* (≥ 90% match with no gaps) and *medium* (≥ 75% match with up to one gap occurring) stringency. The processed reads were compared to the reference mouse (GRCm38/mm10) or human (GRCh37/hg19) genome sequences using BLAT(9). IS in repeat sequence elements were discarded and only those IS with three or more reads of ≥ 35 bp, or two or more reads of ≥ 50 bp per BLAT hit were further analysed. For each IS, distance to transcription start sites (TSS) and transcription site residency were determined using GREAT(10), QuickMap(11) and the UCSC and ENSEMBL genome browsers.

Assessment of toxicity

Mice were transduced at monthly intervals by nasal instillation with one to four doses (1E8 TU/dose) of rSIV-F/HN-CMV carrying Lux or EGFP reporter gene. Controls included untreated and D-PBS treated mice, as well as mice treated with conventional (CpG containing) luciferase plasmid DNA/GL67A complexes or CpG-free CFTR plasmid pGM169/GL67A complexes which were used in our recently completed Phase IIb multi-dose trial(12). The non-viral formulation was prepared as previously described(13). All mice were culled 24 hr after the last dose. Lung tissue sections were stained with haematoxylin&eosin

and were scored semi-quantitatively. Scoring: 0=no inflammation; >0-0.5: =very few/few foci of inflammation in peribronchial or perivascular walls; 1: =patchy cell infiltrates in bronchial or vascular wall in <30% of the section; 2: = localised cell infiltrates in up to 60% of the section; 3: =cell infiltrates in >60% of the section.

Immunohistochemistry

Lungs were processed, cut and de-paraffinised using standard histological procedures. For antigen retrieval slides were treated with pre-heated 0.1M EDTA for 10 min at 100°C. Details of all antibodies and dilutions used are presented in Table E2. Primary and secondary antibodies were incubated at room temperature for 1hr, 0.1% Triton X-100 in 0.1M PBS was used for all dilutions and washing steps. After the final washes sections were mounted with ProLong Gold antifade medium with DAPI (Molecular Probes, Molecular Probes, Life technology, Eugene, Or, USA). Images were generated using a Zeiss LSM-510 inverted confocal microscope (Zeiss, Jena, Germany) using a 40x or 63x oil objective (1.4 NA). The AlexaFluor 594 was excited with the HeNe543 laser. The emission signal was filtered by a 595 nm long pass filter. AlexaFluor 488 was excited with 488 nm and detected with a 505-550nm band pass filter. DAPI was excited with a 405 nm laser and the detection range was 420-490 nm. EGFP expressing airway epithelial cells were quantified using a 63x objective and an Axioskop II fluorescent microscope (Zeiss, Germany) to allow a comparatively large number of airways and cells to be quantified. Twenty random airways and approximately 5000 airway cells were assessed per mouse. Airways were selected using DAPI staining rather than antibody staining to avoid bias for highly transduced regions, but quantification was performed visualising EGFP expression. At a 63x magnification individual cells were clearly visible.

Acquired and Pre-existing immunity

a. Neutralising antibodies in mouse serum

Serum of untreated mice and mice transduced with rSIV.F/HN-Lux (1E8 TU/mouse) was collected 28 days vector administration (n=5-6/group). Sample from each group were pooled and an *in vitro* transduction inhibition assay performed as described below.

b. Anti-Human Parainfluenza virus I immunity in mice (passive immunisation)

rSIV.F/HN-CMV-EGFP was first incubated with 1:2 to 1:32 serial dilutions (duplicate samples for each dilution) of purified anti-hPIV1-3 IgG and IgA antibodies (Abcam, Cambridge, UK) and an *in vitro* transduction inhibition assay was performed as described below.

Mice (n=5/group) received human immunoglobulins (Gamunex® 10% IVIg, (Grifols International, S.A., Barcelona, Spain) by intraperitoneal injection (400 μl, 40 mg IVIG) or intranasal instillation (100 μl, 10 mg IVIG) or remained untreated. 24 hr after passive immunisation human IgG levels were quantified in mouse serum using an anti-hPIV1 IgG Elisa kit according to the manufacturer's protocol (Abcam) to confirm that antibody titres are in a range relevant to what is detectable in human serum (see below). Separate cohorts of mice (n=6/group) were treated with IVIg as described above and transduced with rSIV.F/HN-hCEF-EGFPLux (1E8 TU/mice in 100 μl per/animal) by nasal instillation 24 hr after passive immunisation. Control animals received either vector but no IVIg or IVIg but no vector. Luciferase expression in nose and lung was quantified by using BLI 7 and 28 days after transduction. The mice were then culled and luciferase expression was also quantified in nose and lung tissue homogenate. In addition serum was collected to quantify residual levels of human IgG 29 days post IVIg injection. To assess vector toxicity in mice with pre-existing immunity to hPIV1 we also monitored gross behaviour, body temperature and

weight in all groups in the acute phase after vector administration (until day 5) and bodyweight at the end of the experiment (day 28).

c. Anti-Sendai virus immunity in mice

Mice were transduced with two doses (monthly interval) of transmission-incompetent F protein deleted Sendai virus (ΔF/SeV, 1E6 or 17 infectious units (IU) in 100 μl per mouse, n=8/group) by nasal instillation. The SeV virus did not carry a reporter gene (ΔF/SeV-empty) and was produced by DNAVEC Corporation, Tsukuba, Japan as previously described (14). Control animals remained untransduced. Prior to transduction with rSIV.F/HN-hCEF-EGFPLux (1E8 TU/mice in 100 μl per/animal) 1 month after the second SeV transduction we confirmed that SeV transduced mice had generated anti-SeV IgG antibodies in serum and BALF using an anti-SeV IgG Elisa kit according to the manufacturer's protocol (Alpha Diagnostic International Inc., distributed by Source Bioscience Life Sciences, Nottingham, UK). Before nasal instillation the lentivirus was nebulised through the eFlow® mesh nebuliser (Pari Medical Ltd., West Byfleet, UK) and the aerosol was collected to mimic clinical trial conditions as closely as possible. Control animals remained either (i) untransduced, (ii) received two doses of ΔF/SeV-empty and (iii) no lentivirus or (iv) received lentivirus only.

To assess vector toxicity in mice with pre-existing immunity to SeV animals were monitored daily and assessed using a semi-quantitative gross morphological scoring system monitoring activity, general appearance, posture, hydration and respiration over a 3 month period. Food and water consumption, body temperature and body weight were also monitored.

d. Pre-existing anti-Human Parainfluenza I immunity in serum and broncho-alveolar lavage fluid (BALF)

Serum samples were obtained from the Respiratory Biomedical Research Unit (BRU) biobank using the appropriate biobank ethical approval and consent processes. Adult BALF was obtained as previously described(15). Briefly, all samples were collected from subjects undergoing clinically indicated bronchoscopy. A total volume of 240 ml of warmed saline was instilled into a segment of the right middle lobe and fluid retrieved by gentle manual aspiration. Written informed consent was obtained from all subjects and the study was approved by the Local Research Ethics Committee (Ref 10/H0720/12). Parents of children undergoing a clinically indicated bronchoscopy and BAL (as previously described(16) provided informed consent for an aliquot of BALF to be used for research purposes (Ref 10/H0504/9). Post collection aliquots of unfiltered and unprocessed BALF was immediately placed on ice then stored at -80ºC.

An *in vitro* transduction inhibition assay was performed as previously published (17). Briefly, HEK 293T cells were seeded into 24-well plates (4E5 cells/well) and incubated overnight at 37ºC in 5% CO_2. Heat-inactivated serum (30 min at 56ºC) and BALF were serially diluted with D-MEM (Life Technologies, UK) in a total volume of 100 μl and incubated with 100 μl rSIV.F/HN-CMV-EGFP or rSIV.VSV-G-CMV-EGFP for 1 hour at 37ºC in 5% CO_2. The samples were then added to the HEK-293T cells (n=3 wells/sample) and incubated overnight after which 200 μl D-MEM containing 20% FBS (Sigma –Aldrich, UK) were added and incubated for a further 24 hrs. Cells were then trypsinised, resuspended in D-PBS+1% BSA and the % of EGFP positive cells was calculated using a BDTM LRS II flow cytometer (BD, Biosciences, Canada). A minimum of 10,000 cells per well were counted. Controls included untransduced HEK 293T cells (negative control) and cells transduced with virus not incubated with clinical samples (serum free and BALF free positive controls). All data were expressed as a % of serum and BALF free positive controls, as appropriate. The neutralising

antibody titre was defined as the lowest sample dilution where transduction efficiency reached at least 50% of the positive control sample.

Assessment of CFTR function

a. Iodide efflux measurements were carried out as previously described(1) except that A549 cells (adenocarcinoma human alveolar basal epithelial cells) were used instead of HEK293T cells, because preliminary experiments showed that the hCEF promoter leads to higher levels of gene expression in A549 cells (data not shown).

b. CF Organoids

The Ethics Committee of the Erasmus Medical Centre Rotterdam approved this study and informed consent was obtained. Organoids were generated from rectal biopsies after intestinal current measurements for standard care (subject with CF) or for diagnostic purpose (healthy control) and cultured as described previously(18;19). For viral transduction, organoids (passage 30–40) from a 7-day old culture were trypsinized (TrypLE, Gibco) for 5 min at 37 °C and seeded in 96-well culture plates (Nunc) in 4 μl matrigel (Corning) and virus (1:1 v/v virus:matrigel) containing 100-200 single cells and small organoid fragments as described previously(20). These cells were incubated at 37 °C 10 min and immersed in 150 μl medium. The medium was refreshed (250 μl) 2 days after viral transduction. Four days after viral transduction, organoids were incubated for 30 min with 3 μM calcein-green (Invitrogen), stimulated with forskolin (5 μM) and analyzed by confocal live cell microscopy at 37 °C for 120 min (LSM710, Zeiss). The total organoid area (xy plane) increase relative to t = 0 of forskolin treatment per well was quantified using Volocity imaging software (Improvision). Cell debris and unviable structures were manually excluded from image analysis based on criteria described in detail in a standard operating procedure. The area

under the curve (AUC; t =120 min; baseline =100%) was calculated using Graphpad Prism. After forskolin stimulation and confocal analysis, organoids were lysed in Laemmli buffer supplemented with complete protease inhibitor tablets (Roche). Lysates were analyzed by SDS-PAGE and electrophoretically transferred to a polyvinylidene difluoride membrane (Millipore). The membrane was blocked with 5% milk protein in TBST (0.3% Tween, 10 mM Tris pH8 and 150 mM NaCl in H_2O) and probed 3 h at RT with mouse monoclonal E-cadherin-specific (1:10000; DB Biosciences) or CFTR-specific antibodies (450, 570 and 596; 1:3000; Cystic Fibrosis Folding consortium), followed by incubation with HRP-conjugated secondary antibodies and ECL development. Secreted Gaussia luciferase expression was quantified in the medium as previously described(21).

c. Nasal potential difference measurements in CF mice

The nasal epithelium of gut-corrected cystic fibrosis knockout mice(7) was transduced with rSIV.F/HN-hCEF-CFTR and nasal potential difference measurements were performed as previously described(22).

Vector stability in delivery devices

Virus stability was assessed in a range of delivery devices suitable for vector administration to the lung and nose: Polythylene endoscopic wash catheter (PEC, Olympus KeyMed, UK), Trudell AeroProbe ® catheter (Trudell Medical International, Ontario, Canada) and nasal spray devices (GSK parts No. AR5989 30mL bottle/AR9488 30 ml actuator). An rSIV.F/HN-vector expressing a EGFP or Lux reporter gene was passed through the delivery device and re-collected. HEK293T cells were transduced with the processed vector or with non-processed control virus and EGFP or Lux. Expression was quantified 48 hrs post transduction using routine FACS (on average 20,000 cells were counted for each well) or standard

luciferase assays, respectively. Untransduced cells served as negative control. Stability in each delivery device was assessed in at least two independent experiments. Data are expressed as % of non-passaged control.

Statistical Analysis

ANOVA followed by a Bonferroni post-hoc test or Kruskal-Wallis test followed by Dunns-multiple comparison post-hoc test was performed for multiple group comparison after assessing parametric and non-parametric data distribution with the Kolmogorov-Smirnov normality test, respectively. An independent student t-test or a Mann-Whitney test was performed for two group parametric and non-parametric data as appropriate. All analyses were performed using GraphPad Prism4 and the null hypothesis was rejected at $p<0.05$.

Results

Generation of pharmacopoeia-compliant cGMP vector

All producer plasmids were engineered to be pharmacopoeia-compliant by removal of unnecessary base pairs and replacing the ampicillin-resistance gene with the kanamycin-resistance gene (Virus Production paper; in preparation). To distinguish recombinant SIV vectors generated with the pharmacopoeia-compliant producer plasmids from vector configurations published previously, the vectors in this study are designated rSIV.F/HN or rSIV.VSV-G throughout.

Insertion site (IS) analysis

Samples of mouse lung transduced with rSIV.F/HN produced a total of 85 unique IS when filtered with a high stringency filter and 107 when filtered at medium stringency; the corresponding unique IS from murine nose samples were 14 (high stringency) and 12

(medium stringency) (see Table S3 in the online data supplement). The reason for the lower IS retrieval rate from nose compared with lung samples, despite the higher vector copy number (VCN) in the former is unknown; insufficient gDNA samples remained to further investigate this observation. Only two IS (at either stringency) were obtained from human ALI samples, an outcome likely attributable to the low DNA yield from this source. The rejection rate of reads through filtering and removal of repeat sequences was much higher than anticipated: this was mainly due to removal of unacceptably short (< 35 bp) sequences, implying that the size fractionation step to exclude short DNA fragments had been suboptimal (see Table S3 in the online data supplement).

IS from murine lung and nose samples transduced with rSIV.F/HN-hCEF-EGFPlux were mapped onto the mouse karyogram, and the distances to transcriptional start sites (TSS) determined (see Figure S2 in the online data supplement). Positional analysis showed that 73% of total IS were located in transcription units; six in exons and 73 in introns, of which 42% were integrated in intron 2 (data not shown). Frequency analyses calculated by GREAT using regions defined as IS ± 10 kb and IS ± 100 kb showed that 73% and 70% respectively of each were between 5 and 500 kb from TSS (see Figure S2 in the online data supplement). Although there are insufficient IS to draw definitive conclusions, an exploratory ontological survey revealed no preference for integration near oncogenic loci (data not shown).

Efficient IS retrieval was compromised because samples often produced an excess of short sequence reads. In addition, some samples failed to produce enough DNA to perform the LAM-PCR. Future work will concentrate on refining assay sensitivity to maximise retrieval, and systematically analysing transduced mouse airway samples. Using the accumulated IS data, we will also use gene ontological approaches(23) to further estimate the genotoxic risk by investigating to what extent IS appear in proximity to known oncogenic loci.

References

(1) Mitomo K, Griesenbach U, Inoue M, et al. Toward Gene Therapy for Cystic Fibrosis Using a Lentivirus Pseudotyped With Sendai Virus Envelopes. Mol Ther 2010 March 23;18(6):1173-82.

(2) Hyde SC, Pringle IA, Abdullah S, et al. CpG-free plasmids confer reduced inflammation and sustained pulmonary gene expression. Nat Biotechnol 2008 May;26(5):549-51.

(3) Gill DR, Smyth SE, Goddard CA, et al. Increased persistence of lung gene expression using plasmids containing the ubiquitin C or elongation factor 1alpha promoter. Gene Ther 2001 October;8(20):1539-46.

(4) Yanez-Munoz RJ, Balaggan KS, Macneil A, et al. Effective gene therapy with nonintegrating lentiviral vectors. Nat Med 2006 March;12(3):348-53.

(5) Davies LA, Hyde SC, Nunez-Alonso G, et al. The use of CpG-free plasmids to mediate persistent gene expression following repeated aerosol delivery of pDNA/PEI complexes. Biomaterials 2012 August;33(22):5618-27.

(6) Merten OW, Charrier S, Laroudie N, et al. Large-scale manufacture and characterization of a lentiviral vector produced for clinical ex vivo gene therapy application. Hum Gene Ther 2011 March;22(3):343-56.

(7) Zhou L, Dey CR, Wert SE, et al. Correction of lethal intestinal defect in a mouse model of cystic fibrosis by human CFTR. Science 1994 December 9;266(5191):1705-8.

(8) Schmidt M, Schwarzwaelder K, Bartholomae C, et al. High-resolution insertion-site analysis by linear amplification-mediated PCR (LAM-PCR). Nat Methods 2007 December;4(12):1051-7.

(9) Kent WJ. BLAT--the BLAST-like alignment tool. Genome Res 2002 April;12(4):656-64.

(10) McLean CY, Bristor D, Hiller M, et al. GREAT improves functional interpretation of cis-regulatory regions. Nat Biotechnol 2010 May;28(5):495-501.

(11) Appelt JU, Giordano FA, Ecker M, et al. QuickMap: a public tool for large-scale gene therapy vector insertion site mapping and analysis. Gene Ther 2009 July;16(7):885-93.

(12) Alton EW, Armstrong DK, Ashby D, et al. Repeated nebulisation of non-viral CFTR gene therapy in patients with cystic fibrosis: a randomised, double-blind, placebo-controlled, phase 2b trial. Lancet Respir Med 2015 September;3(9):684-91.

(13) Griesenbach U, Sumner-Jones SG, Holder E, et al. Limitations of the murine nose in the development of nonviral airway gene transfer. Am J Respir Cell Mol Biol 2010 July;43(1):46-54.

(14) Hirata T, Iida A, Shiraki-Iida T, et al. An improved method for recovery of F-defective Sendai virus expressing foreign genes from cloned cDNA. J Virol Methods 2002 July;104(2):125-33.

(15) Molyneaux PL, Cox MJ, Willis-Owen SA, et al. The role of bacteria in the pathogenesis and progression of idiopathic pulmonary fibrosis. Am J Respir Crit Care Med 2014 October 15;190(8):906-13.

(16) Stafler P, Davies JC, Balfour-Lynn IM, et al. Bronchoscopy in cystic fibrosis infants diagnosed by newborn screening. Pediatr Pulmonol 2011 July;46(7):696-700.

(17) Calcedo R, Vandenberghe LH, Gao G, et al. Worldwide epidemiology of neutralizing antibodies to adeno-associated viruses. J Infect Dis 2009 February 1;199(3):381-90.

(18) Dekkers JF, Wiegerinck CL, de Jonge HR, et al. A functional CFTR assay using primary cystic fibrosis intestinal organoids. Nat Med 2013 July;19(7):939-45.

(19) Sato T, Stange DE, Ferrante M, et al. Long-term expansion of epithelial organoids from human colon, adenoma, adenocarcinoma, and Barrett's epithelium. Gastroenterology 2011 November;141(5):1762-72.

(20) Vidoviæ D, Carlon MS, da Cunha MF, et al. rAAV-CFTRÄR rescues the cystic fibrosis phenotype in human intestinal organoids and CF mice. Am J Respir Crit Care Med. In press 2015.

(21) Griesenbach U, Vicente CC, Roberts MJ, et al. Secreted Gaussia luciferase as a sensitive reporter gene for in vivo and ex vivo studies of airway gene transfer. Biomaterials 2011 April;32(10):2614-24.

(22) Griesenbach U, Smith SN, Farley R, et al. Validation of Nasal Potential Difference Measurements in Gut-corrected CF Knockout Mice. Am J Respir Cell Mol Biol 2008 May 5;39(4):490-6.

(23) Biffi A, Montini E, Lorioli L, et al. Lentiviral Hematopoietic Stem Cell Gene Therapy Benefits Metachromatic Leukodystrophy. Science 2013 July 11.

(24) Griesenbach U, Inoue M, Meng C, et al. Assessment of F/HN-pseudotyped lentivirus as a clinically relevant vector for lung gene therapy. Am J Respir Crit Care Med 2012 November 1;186(9):846-56.

Supplement Figures

Figure S1: Survival of lentivirus treated mice

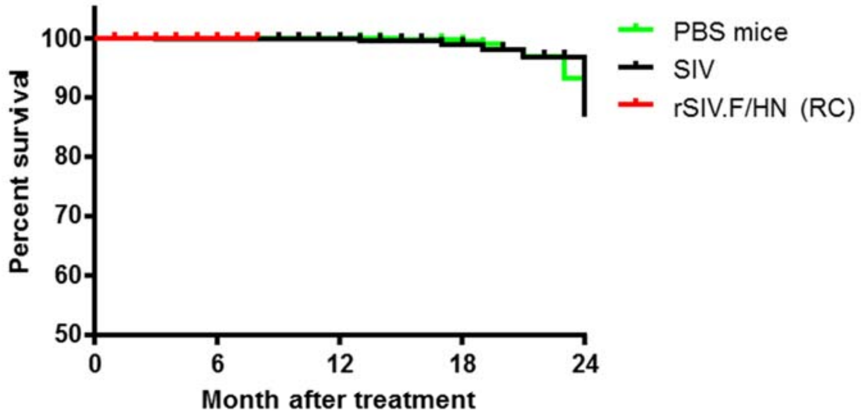

Figure S1

Mice were treated with regulatory compliant (RC) lentiviral vectors (rSIV.F/HN) by nasal sniffing (6-30E7 TU/mouse, n=70 at t=0) and followed for 8 months (n=8 at t=8 months). The remaining mice were culled at interim time-points for other analyses. Survival was compared to previously published data that showed no evidence of chronic toxicity during a 2-year follow-up period (24).

Figure S2. **Distribution of vector integration sites (IS) in rSIV.F/HN-transduced mouse lung and nose.**

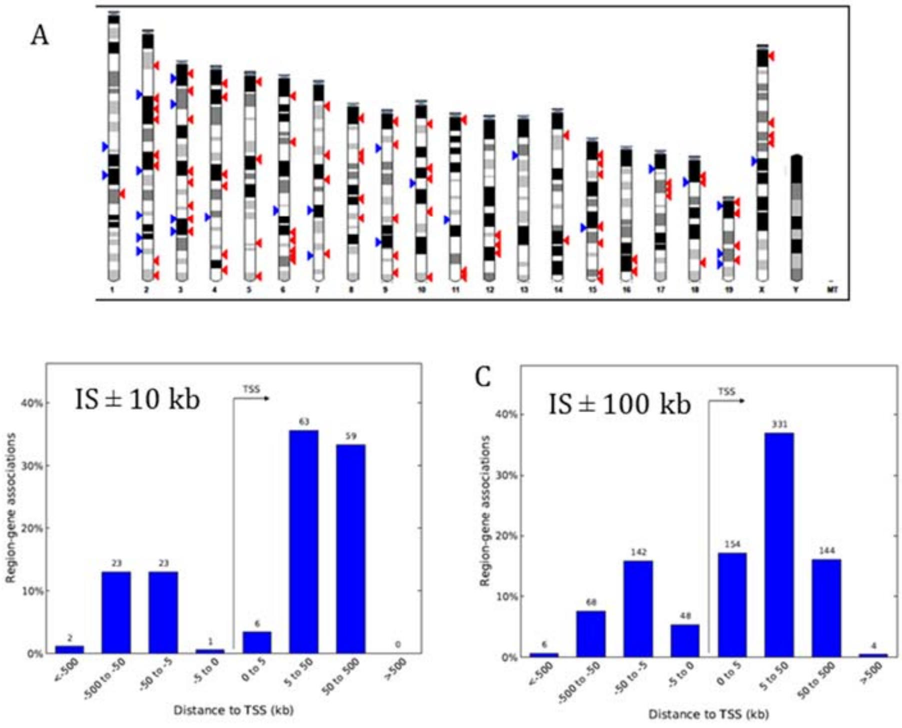

Figure S2

Mice were instilled with rSIV.F/HN vectors expressing either EGFPlux or soCFTR2 under the control of the hCEF promoter (1E8 TU/mouse, n=2-4/group), and culled 5 days after transduction. (**A**) IS plotted on mouse karyogram. High stringency IS are shown as red triangles; additional medium stringency IS are shown as blue triangles. (**B, C**) Distance to nearest transcription start site (TSS) of regions defined as high stringency IS ± flanking sequence: (**B**) regions defined as IS ± 10kb; (**C**) regions defined as IS ± 100 kb.

Figure S3: Neutralising antibodies in mouse serum

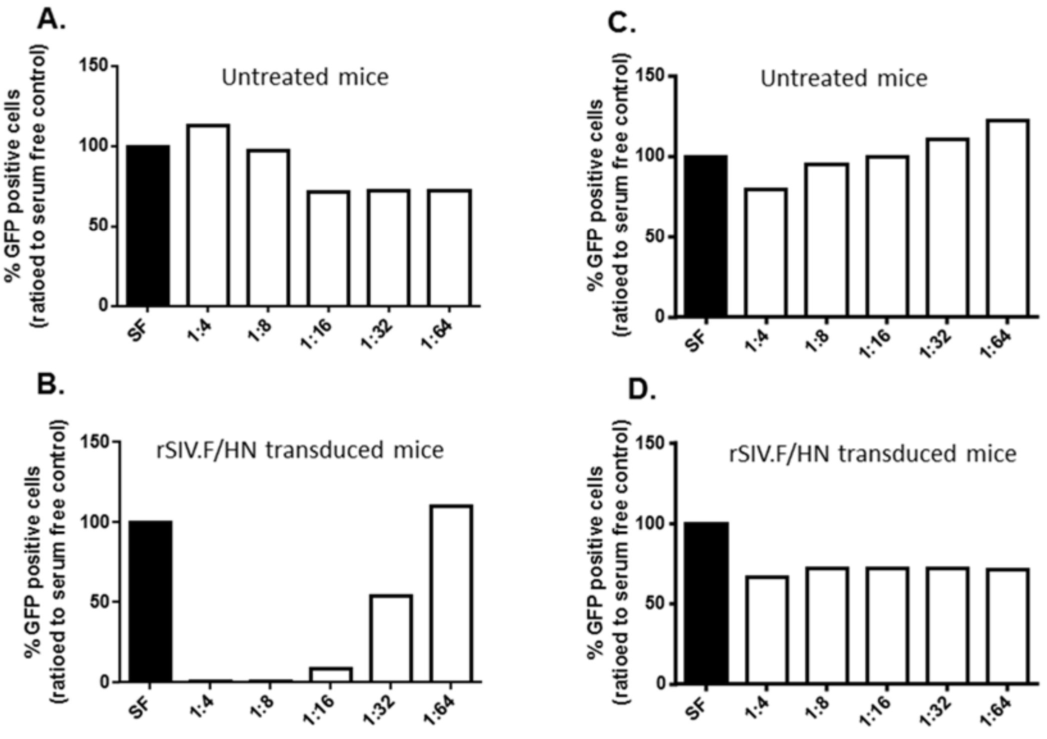

Figure S3

Mice were treated with rSIV.F/HN-CMV-EGFP (1E8 TU/mouse) or remained untreated (n=6/group). 28 days after transduction mice were culled and the serum pooled to perform an *in vitro* transduction inhibition assay in 1:4 to 1:64 serial dilutions of serum (lower dilutions were not feasible due to the small amounts of mouse serum available) to quantify neutralising antibodies. (**A**) Inhibition of rSIV.F/HN transduction in serum of untreated mice, (**B**) inhibition of rSIV.F/HN transduction in serum of transduced mice, (**C**) inhibition of rSIV.VSV-G transduction in serum of untreated mice, (**D**) inhibition of rSIV.VSV-G transduction in serum of transduced mice. Transduction efficiency is presented relative to serum free (SF) controls.

Figure S4: *In vitro* inhibition of rSIV.F/HN by purified hPIV IgG and IgA antibodies

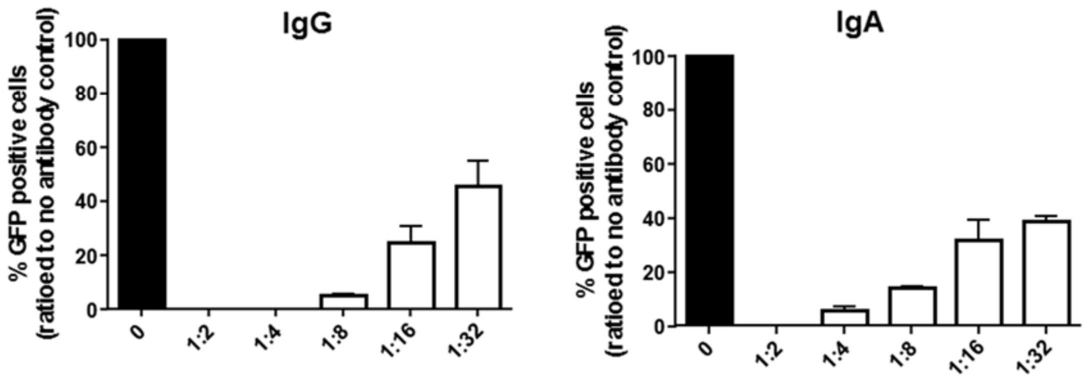

Figure S4

rSIV.F/HN-CMV-EGFP was incubated with 1:2-1:32 serial dilutions of purified IgG and IgA anti-hPIV antibodies and an in vitro transduction inhibition assay was performed. All data are expressed as a ratio of the no-antibody control samples (0). The assay was performed in duplicate.

Figure S5: Anti-hPIV1 IgG in mouse serum and BALF after IVIg injections

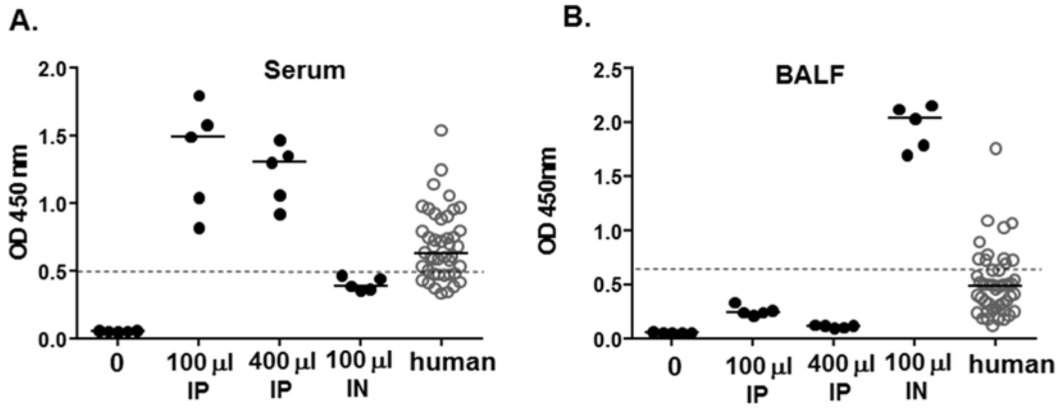

Figure S5

Mice were treated with human immunoglobulin (IVIg) intraperitoneally (IP, 100 or 400 µl) or by nasal instillation (IN, 100 µl). Controls remained untreated (n=5/group). 24 hr after passive immunisation mice were culled and hPIV1 IgG was measured in serum (**A**) and broncho-alveolar lavage fluid (BALF) (**B**). Antibody titres (measured as OD450 nm) were compared to titres measured in human serum (n=43) and BALF (n=47). Each symbol represents one animal/human samples. The horizontal bar indicates the group median. The dotted line indicates the sensitivity limit of the assay. The experiment was performed in duplicate and a representative figure is shown.

Figure S6: Toxicity of rSIV.F/HN transduction in the presence of anti-human hPIV1 antibodies

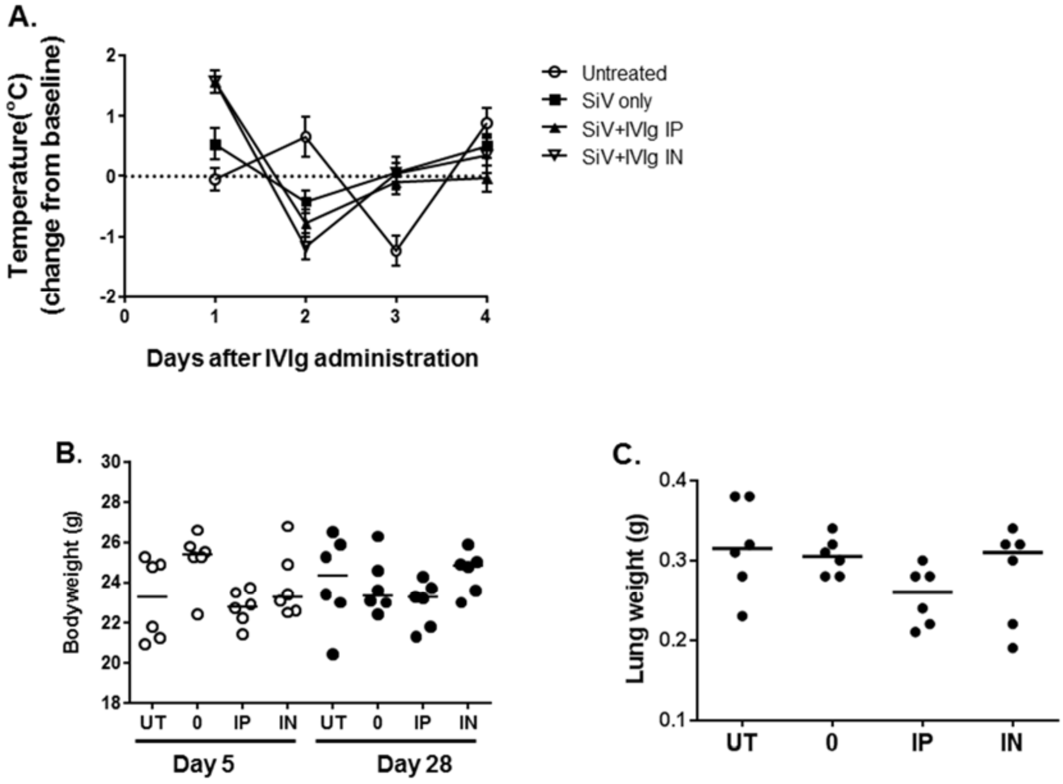

Figure S6

Mice were treated with human immunoglobulin (IVIg) intraperitoneally (IP, 400 μl) or by nasal instillation (IN, 100 μl). Controls did not receive IVIg (n=6/group). 24 hrs after passive immunisation mice were transduced with rSIV.F/HN-hCEF-EGFPLux (1E8 TU/mouse). Control mice remained untreated (UT). (**A**) Body temperature was measured in the acute phase and is expressed as change from pre-treatment baseline. Day 1= one day after IVIg administration, day 2= one day after SIV transduction, day 3= two days after SIV transduction, day 4= three days after SIV transduction. Group mean±SEM are shown (**B**) Body weight was measured 4 and 28 days after SIV transduction, (**C**) Lung weight was determined 28 days after SIV transduction. (**B+C**) Each symbol represents one animal. The horizontal bar shows the group median.

Figure S7: Toxicity of rSIV.F/HN transduction in the presence of anti-Sendai virus antibodies

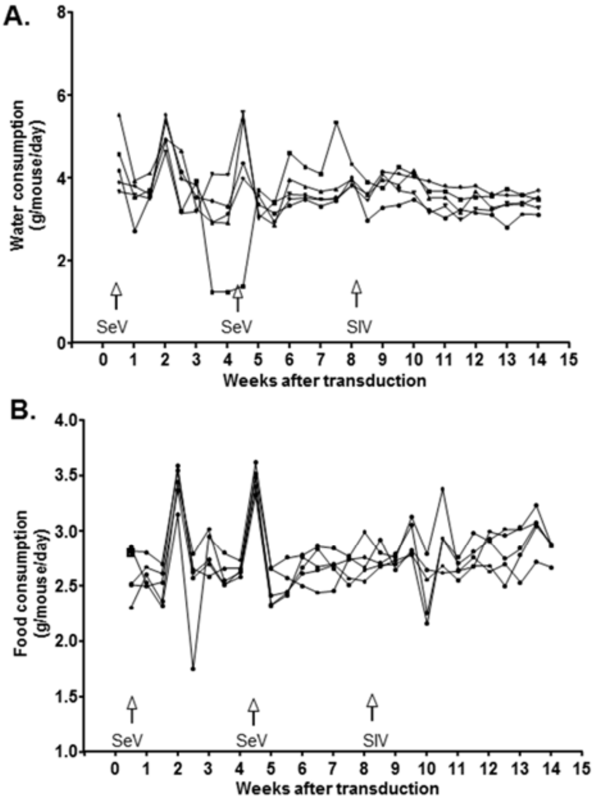

Figure S7

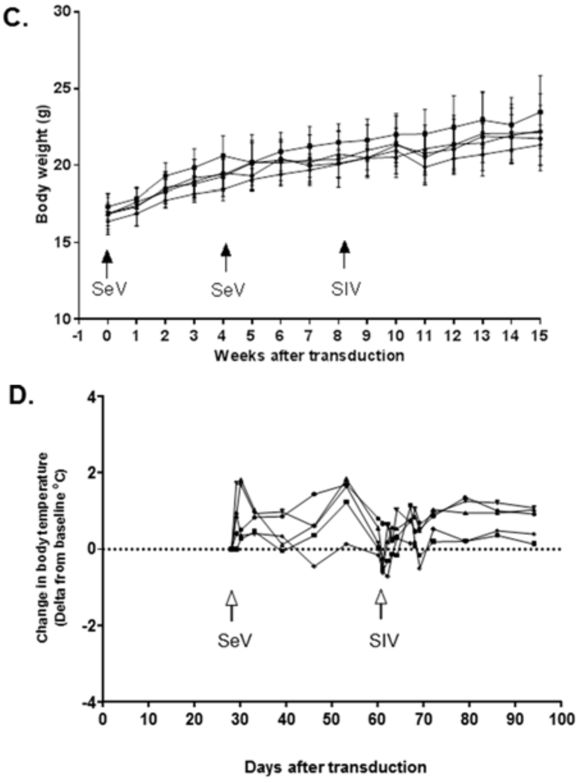

Figure S7 cont'd

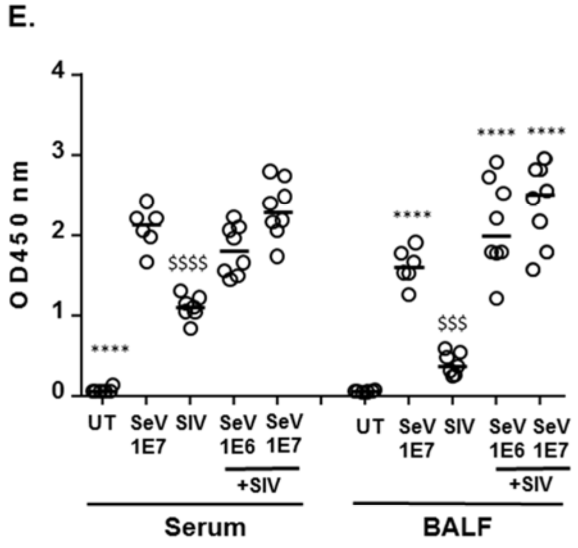

Figure S7 cont'd

Mice were transduced with two doses of ΔF/SeV (1E6 or 1E7 TU/dose) and one dose of rSIV.F/HN (1E8 TU) at approximately monthly intervals by nasal instillation. Control groups remained untransduced (UT) or received two doses of ΔF/SeV (1E7 TU/dose) or one dose of rSIV.F/HN (1.8E8 TU) (n=6-11/group). (**A+B**) Water and food consumption, (**C**) Bodyweight, (**D**) Changes in body temperature (due to technical reasons body temperature was only collected 30 days after the first ΔF/SeV transduction. Circle: =untreated, square:

=SeV only, triangle: =SIV only, inverted triangle: =SeV (1E6TU/dose) + SIV, diamond: =SeV (1E7TU/dose) + SIV, (**E**) Anti-SeV antibody levels were measured in serum and BALF at post-mortem. Each symbol represents one mouse. The median is shown as a

horizontal bar. Serum: ****=p<0.001 compared to all other groups, $$$$=p<0.001 compared to SeV treated groups, BALF: ****=p<0.001 compared to untreated mice, $$$=p<0.005 compared to SeV treated groups (Anova followed by Tukey multiple comparison test).

Figure S8: Neutralising antibodies in human serum

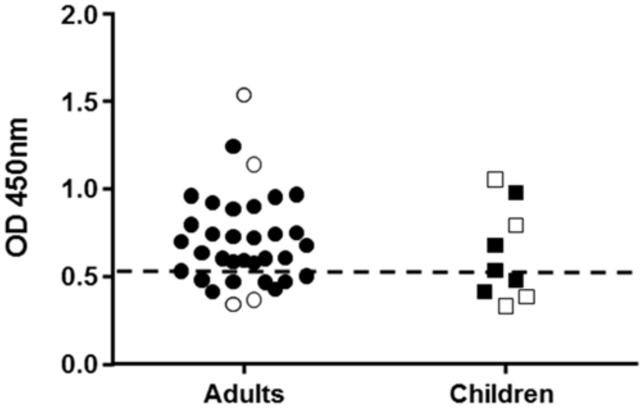

Figure S8 A

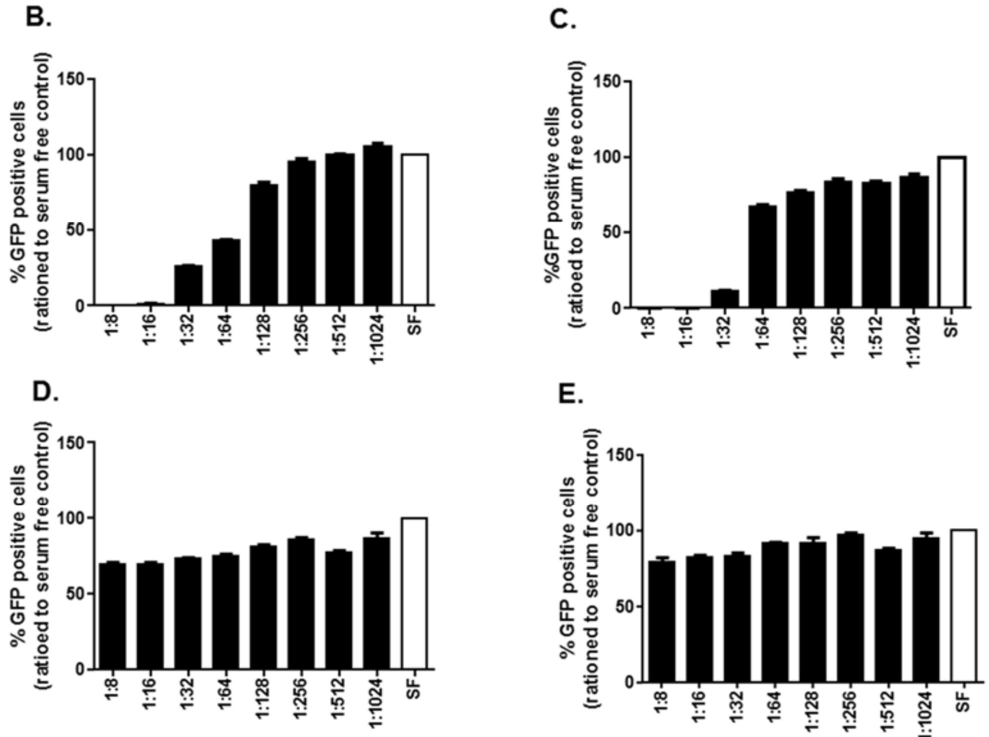

Figure S8

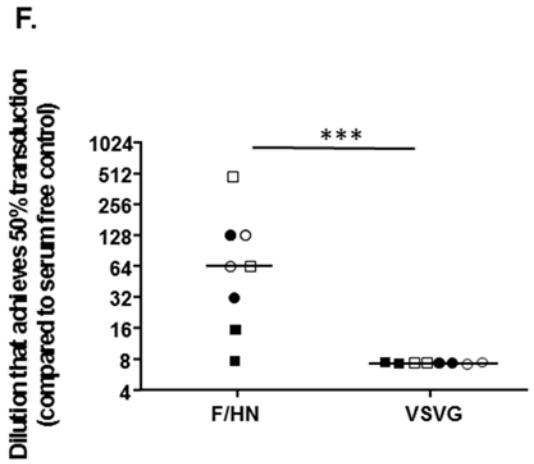

Figure S8 cont'd

Anti-hPIV1 IgG antibodies were quantified in human serum (34 adults (age 21-70) and 9 children (age 2-15) (**A**). The horizontal line indicates the limit of detection for the assay. Open symbols represent samples positive and negative for hPIV1 IgG antibodies that were selected for the transduction inhibition assay. rSIV.F/HN-CMV-EGFP or rSIV.VSV-G - CMV-EGFP were incubated with anti-hPIV1 IgG positive and negative human serum (n=4/group) and an *in vitro* transduction inhibition assay with serial dilutions ranging from 1:8 to 1:1024 was performed to quantify neutralising antibodies (lower dilutions were not feasible due to the small amounts of serum available). Transduction efficiency is presented relative to serum free (SF) controls. Representative results are shown. (**B)** rSIV.F/HN in IgG positive serum, (**C**) rSIV.F/HN in IgG negative serum, (**D**) rSIV.VSV-G in IgG positive serum, (**E**) VSV-G-SIV in IgG negative serum. The same serum samples were used in B+D and C+E. (**F**) Comparison of rSIV.F/HN and VSVG-SIV transduction inhibition. Data are expressed as dilution at which 50% of transduction relative to serum controls is achieved. Open and closed circles show IgG-positive and IgG-negative adults, open and closed squares show IgG-positive and IgG-negative children. ***=p<0.005 (Mann-Whitney test).

Figure S9: Neutralising antibodies in human broncho-alveolar lavage fluid

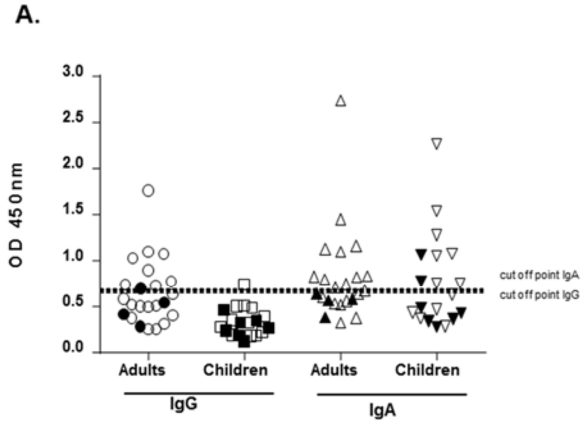

Figure S9

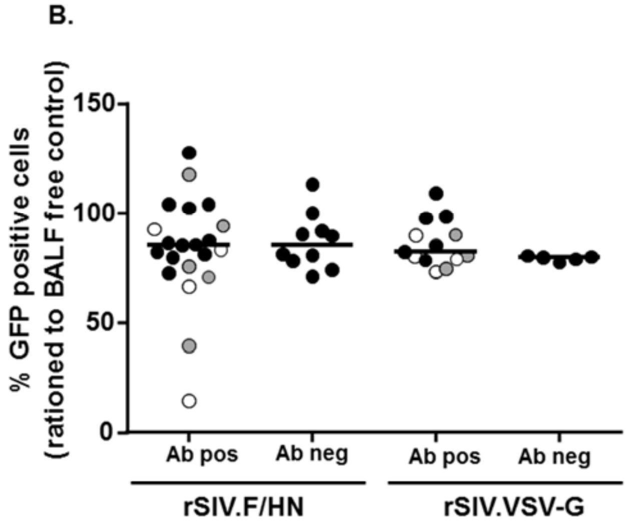

Figure S9 cont'd

Anti-hPIV1 IgG antibodies were quantified in human lavage fluid (n=19 adults (age 18-86), n=12 children (age 2-16)) (**A**). The horizontal lines indicate the limit of detection for the assay. Circles and squares represent serum from adults and children, respectively. Open and closed symbols represent antibody negative and positive samples, respectively. rSIV.F/HN- CMV-EGFP or rSIV.VSV-G-CMV-

EGFP were incubated with anti-hPIV1 IgG- and IgA- positive and negative human BALF and an *in vitro* transduction inhibition assay was performed to quantify neutralising antibodies (**B**). Transduction efficiency is presented relative to BALF free (BF) controls. Each symbol represents one sample. The horizontal line indicates the group mean. To allow robust statistical analysis samples that were positive for either anti-hPIV1 IgG or IgA antibodies were pooled (white symbols=IgG+/IgA+, grey symbols= IgG+/IgA-, black symbols= IgG-/IgA+).

Figure S10: Gaussia luciferase production in intestinal organoids

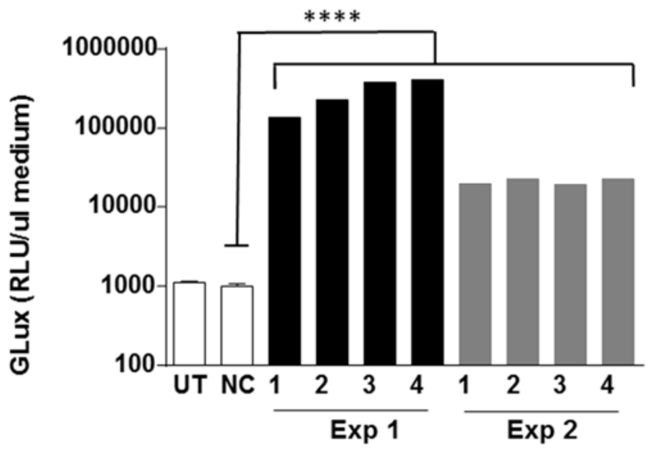

Figure S10

CF intestinal organoids were transduced with rSIV.F/HN-hCEF-soGLux or an irrelevant control virus (NC, n=16) or remained untransduced (UT, n=4). The doses in experiment 1 ranged from 0.45-3.6E7 TU/well and in experiment 2 from 0.06-0.45E7 TU/well (one well/dose). Four days post-transduction Gaussia luciferase (Glux) was measured in the culture medium. Each bar represents one sample except NC and UT where data shows mean±SEM. ****=p<0.0001compared to NC (Unpaired student t-test).

Figure S11: Vector stability in delivery devices

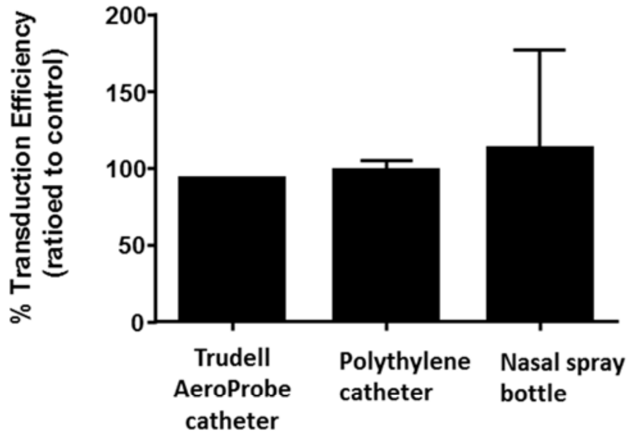

Figure S11

An rSIV.F/HN- vector expressing EGFP was passed through the delivery device and re- collected. A polythylene endoscopic wash catheter, a Trudell AeroProbe ® catheter and a metered-dose nasal spray devices were assessed. HEK293T cells were transduced with the processed vector or with non-processed control virus and EGFP expression was quantified 48 hrs post transduction using routine FACS assays, respectively (n=6 wells/group/experiment). Untransduced cells served as negative control. Stability in each delivery device was assessed in at least two independent experiments. Data are expressed as % of non-passaged control.

Supplement tables

Table S1: Virus titres used in mouse and ALI experiments

Virus	VP/100 μl	TU/100 μl	Relative titre
rSIV.F/HN-CMV-EGFPLux-IC	5E10	1E8	1
rSIV.F/HN-EF1α-EGFPLux-IC	1.8E10	6.1E7	0.616
rSIV.F/HN-hCEF-EGFPLux-IC	5.7E10	1.6E8	1.61
rSIV.F/HN-CMV-EGFPLux-ID	3.6E10	1.2E8	1.15
rSIV.F/HN-hCEF-EGFPLux- ID	7.8E10	2.8E8	2.8

For each vector configuration the maximum feasible volume (100 μl) was used leading to variation in titres applied. For calculation of gene expression the titre was Ratioed relative to that of rSIV.F/HN-CMV-EGFPLux-IC (=1).
VP=virus particles, TU= Transduction Units, IC=Integrase competent, ID=integrase deficient

Table S2: Primary and secondary antibodies used for EGFP detection and cell type characterisation

Cell type	Primary antibody (dilution used)	Supplier of primary antibody	Secondary antibody (dilution used)	Supplier of secondary antibody
Not applicable	Chicken anti-GFP (1:100)	Abcam plc Cambridge, UK	Goat anti-Chicken Alexa Fluor 488 (1:200)	Life Technology Eugene, OR, USA
Not applicable	Rabbit anti-GFP 1/100	Invitrogen Rockford, IL, USA	Goat anti-Rabbit Alexa Fluor 488 (1:200)	Life Technology Eugene, OR, USA
Ciliated airway epithelial cells	Mouse anti β-Tubulin (1:100)	Chemicon Int, Temecula, Ca	Goat anti-Mouse Alexa Fluor 594 (1:200)	Life Technology Eugene, OR, USA
Clara cells	Rabbit anti-Uteroglobin (1:100)	Abcam plc Cambridge, UK	Goat anti-Rabbit Alexa Fluor 594 (1:200)	Life Technology Eugene, OR, USA
Goblet cells	Mouse anti-Mucin 5Ac[45M1] (1: 100)	Abcam plc Cambridge, UK	Goat anti-Mouse Alexa Fluor 594 (1:200)	Life Technology Eugene, OR, USA
Basal cells	Rabbit anti-Cytokeratin 5 (1:100)	Abcam plc Cambridge, UK	Goat anti-Rabbit Alexa Fluor 594 (1:200)	Life Technology Eugene, OR, USA
Type 1 pneumocytes	Hamster anti-Podoplanin (1: 100)	Santa Cruz Biotechnology, Inc Dallas, Texas, USA	Goat anti-Hamster Alexa Fluor 594 (1:200)	Life Technology Eugene, OR ,USA
Type 2 pneumocytes	Rabbit anti-Surfactant protein C (1:100)	Millipore, Temecula, CA	Goat anti-Rabbit Alexa Fluor 594 (1:200)	Life Technology Eugene, OR, USA
Pulmonary macrophages	Rat anti-Mouse F4/80 (1:50)	Serotec, Kidlington, UK	Goat anti-Rat Alexa Fluor 594 (1:200)	Life Technology Eugene, OR, USA

Table S3: Compilation of mouse lung, mouse nose and human ALI samples subject to IS analysis

rSIV.F/HN transgene[1]	Sampling site	No of samples	Input DNA per sample (ng)	Total no of reads[2]	High stringency reads[3]	Medium stringency reads[4]	VCN/cell	High stringency IS[5]	Medium stringency IS[6]
hCEF-EGFPLux	mouse lung	4	9750-14250	405995	11475	25006	0.01-0.05	82	104
hCEF-CFTR	mouse lung	2	9750-14250	21273	49	63	0.01-0.05	3	3
hCEF-EGFPLux	mouse nose	3	375-750	54922	566	847	0.05-0.1	12	10
hCEF-CFTR	mouse nose	2	375-750	98373	131	137	0.05-0.1	2	2
hCEF-CFTR	hALI	2	157-232	49806	48	50	0.03-0.08	2	2

[1]Genotype of rSIV.F/HN vector used in initial transduction
[2]Raw Ion Torrent sequencing reads
[3]High stringency reads remaining after stripping primer, linker, vector and genomic repeat sequences
[4]Medium stringency reads remaining after stripping primer, linker, vector and genomic repeat sequences
[5]Unique high stringency IS remaining after quality control filtering
[6]Unique medium stringency IS remaining after quality control filtering
VCN: Vector Copy Number
